# Cetirizine for the treatment of allergic diseases in children: A systematic review and meta-analysis

**DOI:** 10.3389/fped.2022.940213

**Published:** 2022-08-25

**Authors:** Pengxiang Zhou, Qiong Jia, Zhenhuan Wang, Rongsheng Zhao, Wei Zhou

**Affiliations:** ^1^Department of Pharmacy, Peking University Third Hospital, Beijing, China; ^2^Institute for Drug Evaluation, Peking University Health Science Center, Beijing, China; ^3^Department of Pediatrics, Peking University Third Hospital, Beijing, China; ^4^Department of Pharmacy, First Hospital of Tsinghua University, Beijing, China

**Keywords:** cetirizine, children, allergic diseases, systematic review, meta-analysis

## Abstract

**Objective:**

The global prevalence of allergic diseases has led to a negative and extensive impact on the health and lives of a large population of children. This study investigates the efficacy, acceptability, and safety of cetirizine (CTZ) for treating allergic diseases in children and provides evidence-based assertions for decision-making.

**Methods:**

PubMed, Embase, the Cochrane Library, World Health Organization International Clinical Trials Registry Platform, ClinicalTrials.gov, and the European Union Clinical Trials Register were systematically searched from inception to April 21, 2022. Randomized controlled trials (RCTs) or quasi-RCTs of children with allergic diseases receiving CTZ compared with those receiving placebo or other drugs were included without language limitations. Two investigators independently identified articles, extracted data, conducted meta-analyses, assessed the Cochrane risk of bias of individual studies, and evaluated the evidence certainty using the Grading of Recommendations Assessment, Development, and Evaluation approach; any discrepancies were resolved by consulting with a third investigator. Primary outcomes included scales that evaluated the recovery of allergic conditions in AR, such as the total symptom score (TSS). Secondary outcomes included laboratory test changes, safety (adverse events, AEs), and quality of life (QOL). Data were pooled using the Cochrane Review Manager 5.4, and a fixed-effects model was used if heterogeneity was evaluated as low (*I*^2^ < 50%); otherwise, a random-effects model was adopted.

**Results:**

A total of 22 studies (5,867 patients) were ultimately included [eight with perennial AR, six with seasonal AR, four with atopic dermatitis (AD), and four with other allergic diseases], most of which had a low or unclear risk of bias. Moderate certainty evidence showed that CTZ was found to benefit allergic symptom control [mean difference (MD) of TSS at 1 week: MD, –0.32 (–0.52, –0.12); at 2 weeks: MD, –0.25 (–0.35, –0.14); at 4 weeks: MD, –4.07 (–4.71, –3.43); at 8 weeks: MD, –4.22 (–4.73, –3.72); at 12 weeks: MD, –5.63 (–6.14, –5.13); all *P*-values were less than 0.05] and QOL [at 12 weeks: MD, –23.16 (–26.92, –19.39); *P* < 0.00001] in children with AR. It had similar efficacy compared with other antihistamines (AHs) or montelukast, without showing better control of AD severity in children. Moderate-to-low certainty evidence demonstrated that CTZ was well tolerated and did not increase the risk of severe and overall AEs, cardiotoxicity, damage to the central nervous and digestive systems, or other systems in children, except for the risk of somnolence [risk ratio, 1.62 (1.02, 2.57); *P* = 0.04, compared with placebo].

**Conclusion:**

Moderate-to-low certainty evidence revealed that CTZ could improve clinical improvement and QOL in children with AR and have comparable efficacy with other AHs. CTZ is well tolerated in the pediatric population, except for an increased risk of somnolence.

**Systematic review registration:**

[https://www.crd.york.ac.uk/PROSPERO/], identifier [CRD42021262767].

## Introduction

The most prevalent childhood allergic diseases worldwide include allergic rhinitis (AR), allergic rhino-conjunctivitis, urticaria, asthma, and atopic dermatitis (AD), leading to a negative and extensive impact on the health and lives of a large population of children (1–4). Controlling allergic symptoms in children is of great significance, especially after the global coronavirus disease 2019 (COVID-19) pandemic in 2020. H_1_-antihistamines (H_1_-AHs) play a critical role in controlling allergic symptoms, and cetirizine (CTZ) is one of the most commonly administered second-generation H_1_-AHs in children (5).

Previous results from several clinical trials have reported the efficacy and quality of life (QOL) of CTZ in children with seasonal allergic rhinitis (SAR), chronic spontaneous urticaria, and allergic asthma (6). However, central nervous system (CNS) adverse events (AEs) are increasingly observed in clinical practice and frequently reported (7, 8). The use of CTZ in children was extrapolated from adult patients rather than from direct evidence in children (9). Therefore, the application of CTZ in children, based on direct childhood evidence, remain controversial. Physicians, pharmacists, and patients’ parents express widespread concerns about the efficacy compared with other AHs as well as the long-term safety of CTZ in allergic symptom control.

Unfortunately, except for a narrative description conducted in adults, adolescents, and children with AR (10), there lacks systematic reviews or meta-analyses comprehensively synthesizing remarkable evidence of CTZ application in children with allergic diseases. We, therefore, conducted a systematic review of randomized controlled trials (RCTs) to investigate the benefits and side effects of CTZ in pediatric allergic diseases to fill this gap in knowledge.

## Methods

We conducted a systematic review and meta-analysis of RCTs or quasi-RCTs according to the Preferred Reporting Items for Systematic reviews and Meta-Analyses (PRISMA) reporting guidelines (11) (Supplementary File 1). The study protocol is registered and accessible in PROSPERO (CRD42021262767^1^), and we updated the search dates and the literature compared to the original protocol.

### Data sources and searches

PubMed, Embase, Cochrane Central Register of Controlled Trials (CENTRAL), International Clinical Trials Registry Platform (ICTRP),^2^ ClinicalTrials.gov,^3^ and the European Union Clinical Trials Register^4^ were searched from inception to April 21, 2022. The search strategy was developed and discussed by the review team and consisted of three parts: CTZ, pediatric population, and RCTs using medical subject heading terms, Emtree headings, and text words (Supplementary File 2). The search was limited to human trials without language limitations or years of publication. We also manually searched the reference lists of included studies and previous review articles.

### Eligibility criteria

We sought RCTs or quasi-RCTs that examined the efficacy and safety of CTZ compared with placebo or other drugs for treating allergic diseases, including AR, allergic rhino-conjunctivitis, and urticaria, in children (< 18 years). The dosage forms, treatment duration, and types of allergic diseases were not restricted. The primary outcome of interest was the total symptom score (TSS), scoring atopic dermatitis (SCORAD) (12), and other scales that evaluated the recovery of allergic conditions; secondary outcomes included laboratory tests [total immunoglobulin E (IgE) level, serum eosinophil cationic protein (ECP) values, and total peripheral blood eosinophil counts], Pediatric Rhino-conjunctivitis Quality of Life Questionnaire (PRQLQ) score, and AEs rate. Conference abstracts, editorials, letters, short communications, and publications without peer review were excluded.

### Study selection and data extraction

Two investigators (PZ and QJ) independently screened and assessed the titles, abstracts, and full texts of eligible studies after removing duplicated records; discrepancies were resolved by consulting with a third investigator (WZ or RZ). Using a pre-specified method, two investigators (PZ and QJ) independently extracted data on baseline characteristics, including authors, publication years, countries, number of study centers, allergic diseases, age, study samples, sex, treatment duration, interventions, comparisons, efficacy, and safety outcomes from individual studies. Discrepancies were resolved by consulting with a third investigator (ZW).

### Risk of bias and certainty of evidence

Pairs of independent reviewers assessed the potential risk of bias of individual RCTs, as recommended by Cochrane Collaboration (version 1), based on the following domains: random sequence generation, allocation concealment, blinding, attribution, selective reporting, and other bias (13). Furthermore, the overall quality of evidence for each clinical outcome was evaluated using the Grading of Recommendations Assessment, Development, and Evaluation (GRADE) approach (14). RCT certainty was initially classified as high and was downgraded to moderate, low, or very low certainty if serious flaws were identified in the domains of risk of bias, indirectness of evidence, inconsistency, imprecision, and publication bias. Any discrepancies were resolved by arbitration with a third reviewer (WZ or RZ).

### Data synthesis and analysis

Meta-analyses were conducted separately for each outcome for continuous and binary variables using Review Manager version 5.4 (The Cochrane Collaboration, London, England). Continuous outcomes were expressed as the mean difference (MD) and standardized difference (SD) between groups with 95% confidence intervals (95% CIs). Binary outcomes are presented as relative risk (RR) with 95% CIs.

Heterogeneity among studies was calculated using the chi-square (χ^2^) and *I*^2^ statistics. The fixed-effects model was used for meta-analyses if *I*^2^ < 50% and *P*-value ≥ 0.1. If *I*^2^ ≥ 50%, potential clinical or methodological causes of high heterogeneity were first analyzed, and a random-effects model was used in the forest plots. A two-tailed *P*-value < 0.05 was considered statistically significant. A descriptive analysis was performed if there was insufficient data or high heterogeneity to conduct the meta-analysis.

Subgroup analyses were planned for allergic diseases, comparisons (placebo, other AHs, or montelukast), and treatment durations (1, 2, 4, 8, or 12 weeks). Sensitivity analyses were performed by excluding studies that were evaluated as having a high risk of bias or dominant causes involving heterogeneity. In addition, funnel plots were performed to justify publication bias for the meta-analyses.

## Results

**FIGURE 1:**
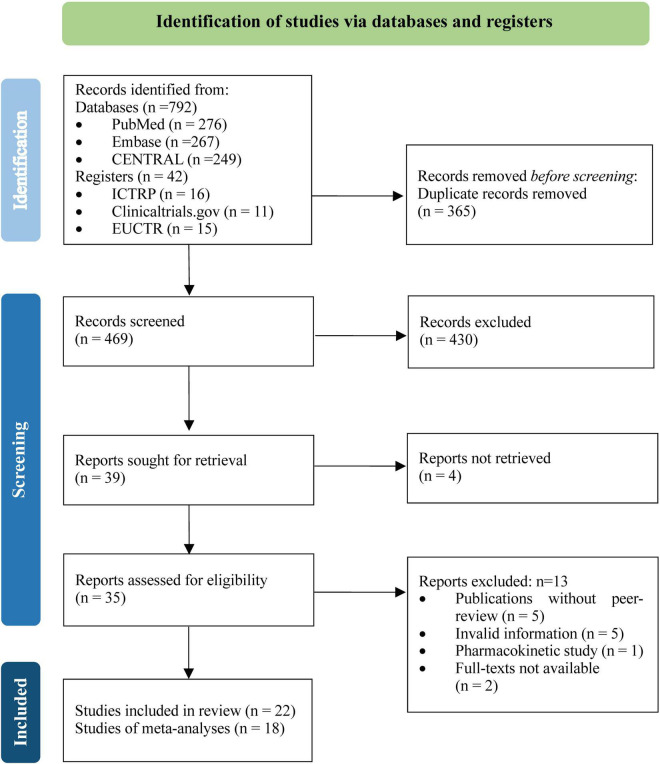
PRISMA flowchart.

A total of 834 unique records were identified from the literature searches, and 469 were screened for titles, abstracts, and full texts after removing duplicates. Twenty two RCTs (15–36), involving 5,867 participants, met the inclusion criteria, of which 18 RCTs (15, 17–30, 32, 33, 35) contributed data to the meta-analyses (Figure 1).

### Study characteristics

The studies were conducted in the United States, European countries, Canada, China, Taiwan, Germany, Netherlands, Singapore, Belgium, and Italy. Eight studies investigated participants diagnosed with perennial allergic rhinitis (PAR), and six studies investigated participants diagnosed with SAR. Four studies were conducted on patients with AD. Only one study reported seasonal allergic rhino-conjunctivitis, mite allergy, chronic allergic rhinitis, and disorders with H_1_-AHs treatment. Furthermore, CTZ was identified as a trial intervention in all eligible studies, and the control groups included ketotifen, chlorpheniramine, terfenadine, loratadine, levocetirizine, montelukast, and placebo (Table 1).

**TABLE 1:** Baseline characteristics of included trials.

Authors and publication years	Countries/ Number of study centers	Allergic disease	Age (years, mean ± SD)	Study samples (T/C)	Gender (T/C, F)	Treatment durations	Interventions, comparisons	Efficacy outcomes	Safety outcomes
Chen (15)	China-Taiwan/NA	PAR	4.53 ± 0.91; 4.49 ± 1.09; 4.36 ± 0.87	Cetirizine/ montelukast/ placebo: 20/20/20	Cetirizine/ montelukast/ placebo: 40.0%/45.0%/ 55.0%	12 weeks	T: cetirizine 5 mg/d; C: montelukast 4 mg/d; placebo (glucose) 5 mg/d	TSS; serum total IgE; ECP values; blood eosinophil counts; PRQLQ score	Somnolence
Delgado (16)	Brazil/single	PAR	7.2/8.4/ 7.2/9.3	20/20/ 20/20	50.0%	14 days	Cetirizine: > 30 kg 10 mg, qd; Terfenadine: 1 mg/kg, bid; Astemizole: 0.2 mg/kg, qd; Loratadine: < 30 kg 5 mg, qd; ≥ 30 kg 10 mg, qd	NA	Cardiotoxicity
Hsieh (17)	China-Taiwan/NA	PAR	8.05 ± 2.39/ 8.20 ± 1.96/ 8.05 ± 1.82	20/20/20	40.0%/35.0%/ 45.0%	12 weeks	T: cetirizine 10 mg/d; C: montelukast 5 mg/d; placebo	TSS; serum total IgE; ECP values; blood eosinophil counts; PRQLQ score	Overall AEs; somnolence; headache; fatigue
Jobst (18)	Netherlands-1/Germany-34	PAR	8.6 ± 1.8/ 9.2 ± 1.9/ 9.3 ± 1.8/ 8.9 ± 1.8	84/85/ 75/83	45.2%/29.4%/ 42.1%/45.8%	14 days	T: cetirizine 2.5 mg, 5 mg, 10 mg; C: placebo	TSS	SAE; overall AEs
Lai (19)	China-Taiwan/NA	PAR	8.16 ± 2.41/ 8.33 ± 2.03/ 7.44 ± 1.41/ 8.31 ± 1.92	19/18/ 16/16	58.7%/56.6%/ 56.2%/56.2%	3 months	T: cetirizine 10 mg/d; C: ketotifen 1 mg, bid; placebo	TSS; serum total IgE; ECP values; blood eosinophil counts; PRQLQ score	Somnolence; headache; fatigue; nausea
Lee (20)	China-Taiwan/NA	PAR	8.19 ± 2.15/ 8.79 ± 1.61/ 8.12 ± 1.68	26/ 24/24	42.3%/37.5%/ 45.8%	12 weeks	T: cetirizine 10 mg/d; C: levocetirizine 5 mg/d; placebo	TSS; serum total IgE; ECP values; blood eosinophil counts; PRQLQ score	Somnolence; fatigue
Ng (21)	Singapore/1	PAR	9.87 ± 1.85	24	41.7%	Single dose	T: cetirizine 10 mg, qd C: chlorpheniramine 4 mg, qd; placebo, qd	NA	Central nervous system AEs
Sienra-monge (22)	Mexico/1	PAR	4.3 ± 1.2/ 4.4 ± 1.1	40/40	40.0%/35.0%	28 days	T: cetirizine 0.2 mg/kg, qd; C: loratadine 0.2 mg/kg, qd	TSS	Somnolence
Allegra (23)	NA/multicenter	SAR	4.0 ± 1.0/ 4.3 ± 1.2	54/53	33.3%/28.3%	2 weeks	T: cetirizine 5 mg/d; C: placebo	TSS	Overall AEs; somnolence; insomnia; headache; abdominal pain; diarrhea; vomiting; increased appetite; nervousness
Nayak (24)	United States/77	SAR	8.6 ± 1.7/ 8.9 ± 1.6/8.9 ± 1.6	228/220/229	42.5%/42.2%/ 46.3%	2 weeks	T: cetirizine 10 mg, qd; C: loratadine 10 mg, qd; placebo, qd	TSS	Somnolence; headache; nausea; vomiting
Pearlman (25)	United States/12	SAR	NA	69/70/66	NA	4 weeks	T: cetirizine 5 mg, qd; cetirizine 10 mg, qd; C: placebo, qd	TSS	Cardiotoxicity; headache; abdominal pain
Tinkelman (26)	NA/4	SAR	8.6/9.1/8.7	62/61/63	35.5%/29.5%/30.2%	2 weeks	T: cetirizine 5–10 mg/d, qd or bid C: chlorpheniramine 2 mg, tid	TSS	Somnolence; headache; fatigue; nausea; abdominal pain
Winder (27)	United States/12	SAR	9.90/8.63/8.83	69/70/68	33.3%/27.1%/36.8%	4 weeks	T: cetirizine 5 mg, qd; cetirizine 10 mg, qd; C: placebo, qd	NA	Cardiotoxicity; headache; abdominal pain
Segal (28)	United States/multicenter	SAR	9.21 ± 1.42/ 9.11 ± 1.47	81/83	35.8%/37.4%	2 weeks	T: cetirizine 5 mg (< 25 kg) or cetirizine 10 mg (≥ 25 kg), qd; C: placebo, qd	TSS	Overall AEs; somnolence; headache; nausea; abdominal pain; nervousness
Baelde (29)	Belgium/multicenter	Chronic AR	8.8 ± 2.1/ 8.5 ± 2.1/ 8.6 ± 2.4	46/46/46	30.4%/30.4%/39.1%	2 weeks	T: cetirizine 2.5 mg, bid; cetirizine 5 mg, bid; C: placebo	TSS	Overall AEs; insomnia; headache; fatigue; abdominal pain
Simons (30)	United States/16	Disorders with H_1_-antihistamine treatment	Boys: 8.5 (6.0–11.0)/8.0 (6.0–11.0); Girls: 7.9 (6.0–11.0)/7.2 (5.0–11.0)	42/43	50.0%/53.49%	1 week	T: cetirizine 0.25 mg/kg, q12h C: placebo, q12h	NA	Overall AEs; somnolence; insomnia; diarrhea; nervousness
Diepgen (31)	12 European countries and Canada	AD	16.8 ± 4.2/ 17.2 ± 4.1 (months)	398/397 (ITT)	38.2%/37.5%	18 months	T: cetirizine 0.25 mg/kg, tid; C: placebo	SCORAD; other oral AHs use rate; the development of urticaria rate	NA
Simons (32)	12 European countries and Canada	AD	16.8 ± 4.1/ 17.2 ± 4.1 (months)	399/396 (ITT)	38.1%/37.6%	18 months	T: cetirizine 0.25 mg/kg, q12h; C: placebo, q12h	NA	SAE; somnolence; insomnia; fatigue; increased appetite; nervousness
Wahn (33)	12 European countries and Canada	AD	16.8 ± 4.2/ 17.2 ± 4.1 (months)	398/397 (ITT)	38.2%/37.5%	18 months	T: cetirizine 0.25 mg/kg, q12h; C: placebo, q12h	SCORAD	Cardiotoxicity; abdominal pain
Warner (34)	12 European countries and Canada	AD	16.8 ± 4.2/ 17.2 ± 4.1 (months)	398/397 (ITT)	38.2%/37.5%	18 months	T: cetirizine 0.25 mg/kg, q12h; C: placebo, q12h	SCORAD; other oral AHs use rate; the development of asthma rate	NA
Masi (35)	Italy/10	Seasonal allergic rhino-conjunctivitis	10.1 ± 0.4/ 10.5 ± 0.5	63/61	39.7%/37.7%	2 weeks	T: cetirizine 10 mg/d; C: placebo	Disease Severity Scores; global evaluation	Overall AEs; somnolence; headache
Ciprandi (36)	Italy/NA	Mite allergy	6.2 (3–10)/6.7 (4–9)	10/10	30.0%/20.0%	6 months	T: cetirizine 5 mg/d; C: placebo	Weekly mean rhinitis symptom scores	NA

T, trial groups; C, control groups; NA, not accessible; AR, allergic rhinitis; PAR, perennial allergic rhinitis; SAR, seasonal allergic rhinitis; AD, atopic dermatitis; ITT, intention-to-treat; TSS, total symptom severity; SCORAD, Scoring Atopic Dermatitis; AHs, H_1_-antihistamines; E, eosinophil cationic protein; PRQLQ, Pediatric Rhino-conjunctivitis Quality of Life Questionnaire; CNS, central nervous system.

### Risk of bias

**FIGURE 2:**
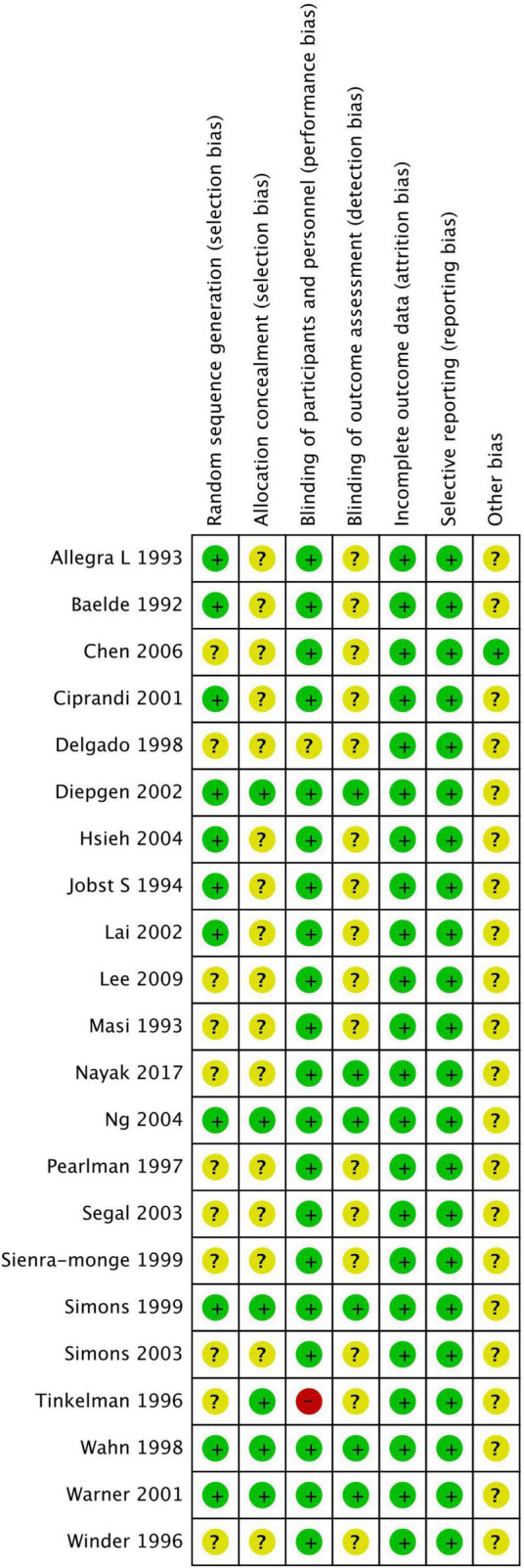
Risk of bias summary.

**FIGURE 3:**
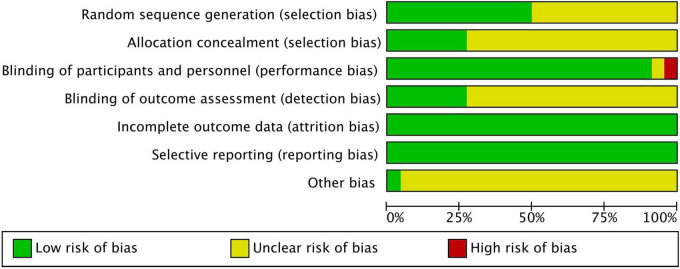
Risk of bias graph.

Figures 2, 3 show the risk of bias assessment results, and Supplementary File 3 presents the details. Half of the trials (*n* = 11) were at low risk of selection bias, reporting a detailed randomization process (*n* = 11). There was inadequate reporting of allocation concealment in 16 trials, which was evaluated as unclear risk of bias. Most of the trials (*n* = 21) were at low or unclear risk of bias in performance, except for one study that was evaluated as having a high risk of bias because of its single-blind design. All studies were at low risk of attribution and reporting biases. Most studies (*n* = 21) did not declare any conflicts of interest.

### Efficacy evaluation

#### Allergic rhinitis

##### Total symptom score

**FIGURE 4:**
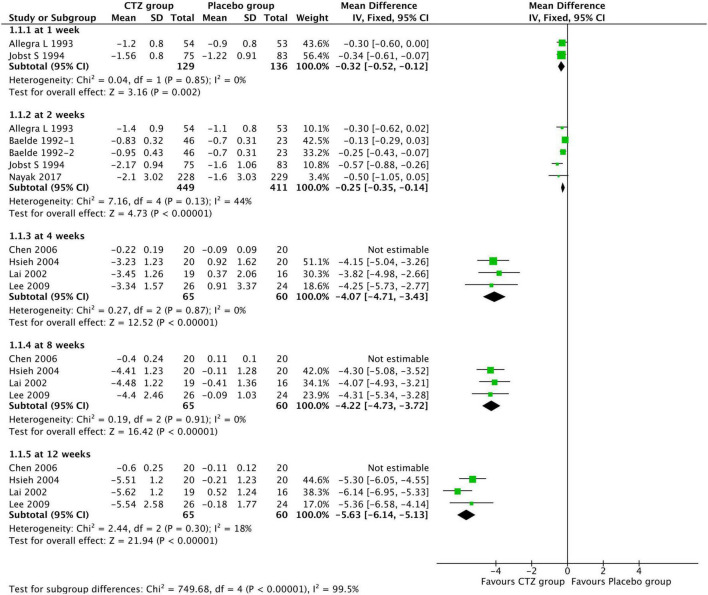
Comparison of cetirizine with placebo for total symptom score in AR children.

Among 12 studies reporting the efficacy of cetirizine for treating AR in children, eight (15, 17–20, 23, 24, 29) used TSS ranging from 0 to 3 to evaluate symptom improvement. Moderate-to-low quality evidence from eight studies showed that compared with placebo, CTZ produced a significantly greater mean TSS reduction at 1 week [two studies with 265 participants; MD, –0.32 (–0.52, –0.12); *I*^2^ = 0%; *P* = 0.002; moderate certainty evidence], 2 weeks [four studies with 860 participants; MD, –0.25 (–0.35, –0.14); *I*^2^ = 44%; *P* < 0.00001; low certainty evidence], 4 weeks [four studies with 125 participants; MD, –4.07 (–4.71, –3.43); *I*^2^ = 0%; *P* < 0.00001; moderate certainty evidence], 8 weeks [four studies with 125 participants; MD, –4.22 (–4.73, –3.72); *I*^2^ = 0%; *P* < 0.00001; moderate certainty evidence], and 12 weeks [four studies with 125 participants; MD, –5.63 (–6.14, –5.13); *I*^2^ = 0%; *P* < 0.00001; moderate certainty evidence] (Figure 4). We did not pooled data from Chen et al. (15) mainly because it might be a source of high heterogeneity.

Furthermore, narrative analyses of the remaining four studies were conducted (22, 25, 26, 28) because of the inconsistency in data form and comparisons. Compared with placebo, two studies (25, 28) reported that 10 mg CTZ daily showed significant improvements in symptom control, and 5 mg CTZ daily produced similar efficacy. Additionally, Tinkelman et al. (26), with high risk of bias, revealed no significant differences in TSS between CTZ administered once or twice daily and chlorpheniramine groups. Sienra et al. (22) reported that CTZ and loratadine improved symptoms, but the differences between the two groups were not significant.

##### Laboratory tests

Cetirizine was found to be associated with lower serum total IgE levels [four studies (15, 17, 19, 20) with 165 participants; MD, –89.75 (–144.78, –34.72); *I*^2^ = 0%; *P* = 0.001], serum ECP values [four studies (15, 17, 19, 20) with 165 participants; MD, –3.81 (–5.00, –2.61); *I*^2^ = 0% *P* < 0.00001], and total peripheral blood eosinophil counts [four studies (15, 17, 19, 20) with 165 participants; MD, –161.93 (–226.09, –97.77); *I*^2^ = 0%; *P* < 0.00001] compared with placebo. When compared with montelukast, CTZ showed similar efficacy in terms of serum total IgE levels [two studies (15, 17) with 85 participants; MD, –39.19 (–127.31, 48.94); *I*^2^ = 0%, *P* = 0.38], serum ECP values [two studies (15, 17) with 85 participants; MD, –0.75 (–4.36, 2.85); *I*^2^ = 0%; *P* = 0.68], and total peripheral blood eosinophil counts [two studies (15, 17) with 85 participants; MD, –50.38 (–162.39, 61.63); *I*^2^ = 0%; *P* = 0.38]. However, CTZ was inconsistent when compared with levocetirizine (20) and ketotifen (19) in laboratory tests; therefore, further investigations should be conducted (Supplementary File 4.1–4.3).

##### Pediatric Rhino-conjunctivitis Quality of Life Questionnaire score

Cetirizine significantly decreased the mean PRQLQ score at 12 weeks after treatment compared with placebo [three studies (15, 19, 20) with 125 participants; MD, –23.16 (–26.92, –19.39); *I*^2^ = 0%; *P* < 0.00001; moderate certainty evidence; narrative analysis from one study (17)]. However, there was no significant difference between cetirizine and other AHs [two studies (19, 20) with 85 participants; MD, –0.88 (–11.99, 10.22); *I*^2^ = 67%; *P* = 0.88; low certainty evidence] and montelukast [one study (15) with 40 participants; MD, –12.00 (–25.68, 1.68); *P* = 0.09] (Supplementary File 4.4).

#### Atopic dermatitis

Diepgen et al. (31) and Warner et al. (34) reported that the severity of AD, as measured by SCORAD, decreased significantly in the CTZ and control groups (*P* < 0.001), without statistical differences between the groups over the 18-month treatment period, except for the severity of eczema. Nevertheless, the rates of taking oral AHs as rescue medication (18.6 vs. 24.9%, *P* = 0.03) and the development of urticaria (5.8 vs. 16.2%, *P* < 0.001) or asthma in infants sensitized to grass pollen and house dust mite were significantly lower in the CTZ group than in the placebo group.

#### Other diseases

For children with pollen-associated rhino-conjunctivitis, Masi et al. (35) reported that CTZ was associated with a significantly greater improvement in allergic symptoms (Disease Severity Score, DSS) compared with placebo evaluated by patients (DSS = 0, *P* = 0.007; DSS ≤ 1, *P* = 0.0001; DSS ≤ 2, *P* = 0.0004) and investigators (after 1 week, *P* = 0.007; after 2 weeks, *P* < 0.001). Global evaluation using the non-stratified Cochrane-Mantel-Haenszel (CHM) test at the end of treatment showed greater improvement with CTZ than with the placebo (*P* < 0.001).

For mite allergies, Ciprandi et al. (36) revealed that the weekly mean rhinitis symptom scores of the CTZ-treated group (5 mg/day) were significantly lower than those of the placebo group (*P* < 0.05). The weekly mean asthma symptom scores were significantly lower in the CTZ-treated group than in the placebo group (*P* < 0.05) for 6 weeks. However, this difference was not significant in the remaining weeks.

### Safety evaluation

#### Severe adverse events and overall adverse events

Very low rates of severe adverse events (SAEs) were reported in all the studies. Only two studies recorded drug-related SAEs, and no statistical difference was found between CTZ and placebo [two studies (18, 32) with 1,122 participants; RR, 0.35 (0.09, 1.36); *I*^2^ = 0%, *P* = 0.13]. In addition, CTZ did not increase the risk of overall AEs compared with placebo [seven studies (17, 18, 23, 28–30, 35) with 893 participants; RR, 1.07 (0.85, 1.34); *I*^2^ = 4%; *P* = 0.58; high certainty evidence] (Supplementary File 5.1).

#### Cardiotoxicity

Based on the descriptive analysis of four studies (16, 25, 27, 33), 5 or 10 mg of CTZ daily did not significantly increase the risk of cardiotoxicity, including QT interval prolongation, compared with placebo.

#### Central nervous system

Pooled results demonstrated that CTZ may be associated with an increased rate of somnolence compared to placebo [ten studies (15, 17, 19, 20, 22–24, 26, 28, 30, 32, 35) with 1,823 participants; RR, 1.62 (1.02, 2.57); *I*^2^ = 9%; *P* = 0.04; moderate certainty evidence]. However, children administered CTZ or other AHs [five studies (19, 20, 22, 24, 26) with 769 participants; RR, 1.61 (0.72, 3.58); *I*^2^ = 0%; *P* = 0.24; moderate certainty evidence], or montelukast [two studies (15, 17) with 80 participants; RR, 3.00 (0.33, 27.69); *I*^2^ = 0%; *P* = 0.33; low certainty evidence] had a similar likelihood of somnolence (Supplementary File 5.2). Furthermore, Ng et al. reported that chlorpheniramine and CTZ could increase P300 latency compared with baseline, but with no significant change in somnolence (21).

In addition, when compared with placebo, CTZ did not increase the incidence of insomnia [four studies (23, 29, 30, 32) with 1,079 participants; RR, 1.04 (0.49, 2.22); *I*^2^ = 55%; *P* = 0.91; moderate certainty evidence], headache [nine studies (17, 19, 23–25, 27–29, 35) with 1,477 participants; RR, 0.84 (0.58, 1.21); *I*^2^ = 0%; *P* = 0.35; moderate certainty evidence], and fatigue [four studies (17, 19, 32) with 1,008 participants; RR, 1.93 (0.84, 4.40); *I*^2^ = 0%; *P* = 0.12; moderate certainty evidence]. Moreover, no significant difference was observed between CTZ and other AHs in the rate of headache [two studies (24, 26) with 634 participants; RR, 0.78 (0.36, 1.71); *I*^2^ = 0%; *P* = 0.53; low certainty evidence], and fatigue [three studies (19, 20, 26) with 241 participants; RR, 0.70 (0.24, 2.02); *I*^2^ = 0%; *P* = 0.51; low certainty evidence]. CTZ had a similar risk of headache as montelukast [one study (17) with 40 participants; RR 1.00 (0.07, 14.90); *P* = 1.00] (Supplementary File 5.3–5.6).

#### Digestive system

Compared with placebo, CTZ was not associated with higher risks of nausea [two studies (19, 28) with 621 participants; RR, 0.96 (0.07, 13.69); *I*^2^ = 51%; *P* = 0.97; low certainty evidence], abdominal pain [six studies (23, 25, 27–29, 33) with 1,616 participants; RR, 1.22 (0.75, 1.99); *I*^2^ = 0%; *P* = 0.42; high certainty evidence], diarrhea [two studies (23, 30) with 192 participants; RR, 1.02 (0.27, 3.93); *I*^2^ = 0%; *P* = 0.97; moderate certainty evidence], vomiting [two studies (23, 24) with 192 participants; RR, 1.00 (0.17, 5.68); *I*^2^ = 0%; *P* = 1.00; moderate certainty evidence], and increased appetite [two studies (23, 32) with 902 participants; RR, 2.31 (0.43, 15.51); *I*^2^ = 0%; *P* = 0.39; moderate certainty evidence]. However, compared with other AHs, CTZ had a risk of nausea [three studies (19, 24, 26) with 669 participants; RR, 0.58 (0.11, 3.18); *I*^2^ = 17%; *P* = 0.53; moderate certainty evidence], abdominal pain [one study (26) with 186 participants; RR, 2.05 (0.60, 7.00); *P* = 0.25], and vomiting [one study (24) with 448 participants; RR, 0.48 (0.09, 2.61); *P* = 0.40] (Supplementary File 5.7–5.10).

#### Other systems

Compared with placebo, CTZ had similar likelihood of nervousness [four studies (23, 28, 30, 32) with 1,151 participants; RR, 0.76 (0.46, 1.26); *I*^2^ = 0%; *P* = 0.28; moderate certainty evidence] (Supplementary File 5.11), tremor [One study (30), 85 participants; RR, 1.02 (0.07, 15.84); *P* = 0.99], irritability [One study (22), 80 participants; RR, 3.00 (0.13, 71.51); *P* = 0.50], hyperkinesia [One study (32), 795 participants; RR, 0.55 (0.19, 1.63); *P* = 0.28], depression [One study (23), 107 participants; RR, 0.33 (0.01, 7.86); *P* = 0.49], respiratory tract [One study (23),107 participants; RR 0.42 (0.11, 1.54); *P* = 0.19], pharyngitis [One study (25), 205 participants; RR, 0.74 (0.34, 1.62); *P* = 0.45], epistaxis [One study (25), 205 participants, RR; 1.58 (0.45, 5.56); *P* = 0.47], rash [One study (28), 164 participants; RR, 7.17 (0.38, 136.66); *P* = 0.19], febrile convulsions [One study (32), 795 participants; RR, 0.50 (0.09, 2.69); *P* = 0.42], and ataxia [One study (32), 795 participants; RR, 0.99 (0.14, 7.01); *P* = 0.99].

## Discussion

### Meaning of this review

This study found moderate certainty evidence that CTZ is well established in reducing symptoms and obtaining a better QOL in children with AR from 1 to 12 weeks and has similar efficacy compared with other AHs or montelukast. CTZ might improve the allergic symptoms of rhino-conjunctivitis but not clinically decrease the severity of AD in children. Furthermore, moderate-to-low certainty evidence showed that CTZ was well tolerated and did not increase the risk of SAEs, overall AEs, cardiotoxicity, CNS (excluding somnolence), digestive system, or other systems in children. Although CTZ is widely used in pediatric clinical practice, there is currently a lack of strong evidence regarding its application in children, with reference to clinical pharmacology data obtained in adults or teenagers (37). To our best knowledge, this review first comprehensively summarizes and updates the evidence for the use of CTZ in childhood allergic diseases.

### Evidence summary and update

Second-generation oral AHs are standard management for histamine-mediated allergic conditions (38), but the efficacy and safety of AHs still lead to widespread concern in children. CTZ has derived from the metabolism of hydroxyzine 30 years ago, with high specificity for the H_1_ receptors and antiallergic properties (6). In this review, pooled results based on RCTs evidence only supported those pediatric patients with AR or other allergic diseases with eczema would benefit from CTZ, and limited data supported the use of CTZ in children with urticaria. Theoretically, CTZ is thought to have a better pharmacokinetic profile, with rapid dose-independent absorption, no clinically relevant accumulation, and a low potential for drug interactions (39). It is only slightly metabolized in the liver and then eliminated by renal excretion (5). Nonetheless, there was no clinical evidence that oral CTZ was more effective than other oral AHs or leukotriene receptor antagonists in this review, which was also demonstrated in the clinical guidelines for pediatric chronic urticaria (40). Therefore, the management of AR or urticaria should be individualized according to the response, compliance, and economic situations of pediatric patients.

Antihistamines are among the most prescribed agents in pediatric care, and there is increasing recognition of the importance of their safety and tolerance profiles. To the best of our knowledge, first-generation AHs can cross the blood-brain barrier, bind to CNS H_1_ receptors, and lead to CNS-related AEs. As a relatively higher and more favorable affinity and selectivity for the H_1_ receptor antagonist, CTZ confers a more potent, faster onset, and longer duration of action (41). However, clinical investigations and spontaneous data analyses indicate that CTZ is also associated with an increased risk of drug-related CNS reactions (7, 8), such as somnolence, which was previously indicated by a safety evaluation of newer-generation AHs (42). In addition, the danger of AEs involving growth impairment or cognitive development is particularly important. Early Treatment of the Atopic Child (ETAC) studies revealed that CTZ did not influence growth, learning skills, and neurologic and behavioral functions (43) in children during an 18-month treatment period. Studies in adults have revealed that AHs might affect psychomotor performance and memory processing speed but not memory or cognitive impairment (44–46). Furthermore, CTZ-related cardiovascular safety events, including torsade de pointes, QT abnormalities, ventricular arrhythmia, and sudden cardiac death or cardiac arrest, have resulted in signals from drug safety databases worldwide (47, 48). Nevertheless, currently available studies in the pediatric population do not support routine electrocardiogram monitoring, except for children with inherited long QT syndrome, cardiovascular disorders, hypokalemia, or hypomagnesemia (49, 50).

### Unanswered questions and future research

Uncertain issues regarding CTZ use in children remain unanswered, and more well-designed controlled trials are needed immediately. First, the guidelines did not agree on the combined use of two AHs in cases of poor reaction to single AHs with standard dosage and treatment duration (51, 52). Therefore, further trials should be carried out to determine whether increasing dosage, combinational use of two AHs, or switching to drugs with different action mechanisms can be prioritized in specific allergic diseases. Second, when patients with recalcitrant urticaria do not respond to conventional doses of CTZ (10 mg daily), doses are increased up to fourfold, in line with an off-label use recommended by current guidelines; further studies are needed to demonstrate its clinical feasibility and safety. A narrative review of a limited sample revealed that 20 mg CTZ daily (twice the recommended dose) might improve clinical efficacy, but 30 mg daily might not yield better control of moderate or severe chronic urticaria (53, 54). Third, further studies should focus on intravenously administered CTZ, which has already been approved to treat acute urticaria in children as young as 6 months of age. A narrative review reported that intravenous CTZ was non-inferior to intravenous diphenhydramine in terms of a 2-h pruritus score and was associated with fewer AEs (55). Finally, besides its relevant anti-allergic activity, studies have revealed that CTZ might have potential anti-inflammatory properties with known or unknown mechanisms, which might be new therapeutic targets for multiple pediatric allergic conditions (56, 57).

### Strengths and weaknesses of this review

This systematic review has several strengths and weaknesses. Highlighting the strengths, the study, which follows the PRISMA reporting guidelines, has been e prospectively registered online. Also, to the best of our knowledge, the current largest sample of this review can provide the best evidence on the efficacy and safety of CTZ, as only RCTs with risk of bias evaluation were included. Moreover, the GRADE approach was used to assess evidence certainty. Regrettably, a critical limitation already cited is the early publication year of the eligible studies, resulting in the inability to include recent data on cetirizine use in children. In addition, we did not analyze the publication bias of certain outcomes, which might be attributed to studies with small sample sizes and insufficient studies (usually less than 10) (58). This was carefully considered in evaluating evidence certainty using the GRADE approach (Supplementary File 6).

In conclusion, this systematic review found moderate-to-low certainty evidence that CTZ could be associated with better clinical improvement and QOL in children with AR compared with placebo and has comparable efficacy with other AHs in children. Although CTZ is well tolerated in the pediatric population, except for an increased risk of somnolence, the combinational and uploading doses of CTZ still require further investigation.

## Data availability statement

The original contributions presented in this study are included in the article/Supplementary material, further inquiries can be directed to the corresponding authors.

## Author contributions

PZ and QJ conducted the study registration, literature selection, review, and data analyses. PZ conducted the literature search. PZ and ZW conducted quality assessment and GRADE evaluation. WZ and RZ provided the pediatric and pharmacological guidance. All authors participated in the research design, contributed to the writing of this manuscript, and approved the final version.

## Footnotes

1
www.crd.york.ac.uk/prospero

2
https://trialsearch.who.int/

3
http://www.clinicaltrials.gov

4
https://www.clinicaltrialsregister.eu

## References

[1] Goncalo M Gimenez-Arnau A Al-Ahmad M Ben-Shoshan M Bernstein JA Ensina LF The global burden of chronic urticaria for the patient and society. *Br J Dermatol.* (2021) 184:226–36. 10.1111/bjd.19561 32956489

[2] Brozek JL Bousquet J Agache I Agarwal A Bachert C Bosnic-Anticevich S Allergic rhinitis and its impact on asthma (ARIA) guidelines-2016 revision. *J Allergy Clin Immunol.* (2017) 140:950–8. 28602936 10.1016/j.jaci.2017.03.050

[3] Blaiss MS Hammerby E Robinson S Kennedy-Martin T Buchs S. The burden of allergic rhinitis and allergic rhinoconjunctivitis on adolescents: a literature review. *Ann Allergy Asthma Immunol.* (2018) 121:43–52e43. 10.1016/j.anai.2018.03.028 29626629

[4] Fishbein AB Silverberg JI Wilson EJ Ong PY. Update on atopic dermatitis: diagnosis, severity assessment, and treatment selection. *J Allergy Clin Immunol Pract.* (2020) 8:91–101. 10.1016/j.jaip.2019.06.044 31474543 PMC7395647

[5] Parisi GF Leonardi S Ciprandi G Corsico A Licari A Miraglia Del Giudice M Cetirizine use in childhood: an update of a friendly 30-year drug. *Clin Mol Allergy.* (2020) 18:2. 10.1186/s12948-020-00118-5 32127782 PMC7043022

[6] Corsico AG Leonardi S Licari A Marseglia G Miraglia Del Giudice M Peroni DG Focus on the cetirizine use in clinical practice: a reappraisal 30 years later. *Multidiscip Respir Med.* (2019) 14:40. 10.4081/mrm.2019.487 31827796 PMC6898951

[7] Motola D Donati M Biagi C Calamelli E Cipriani F Melis M Safety profile of H1-antihistamines in pediatrics: an analysis based on data from VigiBase. *Pharmacoepidemiol Drug Saf.* (2017) 26:1164–71. 10.1002/pds.4246 28653802

[8] Ferrer M Morais-Almeida M Guizova M Khanferyan R. Evaluation of treatment satisfaction in children with allergic disease treated with an antihistamine: an international, non-interventional, retrospective study. *Clin Drug Investig.* (2010) 30:15–34. 10.2165/11530910-000000000-00000 19995095

[9] Nieto A Nieto M Mazon A. The clinical evidence of second-generation H1-antihistamines in the treatment of allergic rhinitis and urticaria in children over 2 years with a special focus on rupatadine. *Expert Opin Pharmacother.* (2021) 22:511–9. 10.1080/14656566.2020.1830970 33198523

[10] Zhang L Cheng L Hong J. The clinical use of cetirizine in the treatment of allergic rhinitis. *Pharmacology.* (2013) 92:14–25. 10.1159/000351843 23867423

[11] Page MJ McKenzie JE Bossuyt PM Boutron I Hoffmann TC Mulrow CD The PRISMA 2020 statement: an updated guideline for reporting systematic reviews. *BMJ.* (2021) 372:n71. 10.1136/bmj.n71 33782057 PMC8005924

[12] Chopra R Vakharia PP Sacotte R Patel N Immaneni S White T Severity strata for eczema area and severity index (EASI), modified EASI, scoring atopic dermatitis (SCORAD), objective SCORAD, atopic dermatitis severity index and body surface area in adolescents and adults with atopic dermatitis. *Br J Dermatol.* (2017) 177:1316–21. 10.1111/bjd.15641 28485036

[13] Higgins JP Altman DG Gotzsche PC Juni P Moher D Oxman AD The Cochrane Collaboration’s tool for assessing risk of bias in randomised trials. *BMJ.* (2011) 343:d5928. 10.1136/bmj.d5928 22008217 PMC3196245

[14] Balshem H Helfand M Schunemann HJ Oxman AD Kunz R Brozek J GRADE guidelines: 3. Rating the quality of evidence. *J Clin Epidemiol.* (2011) 64:401–6. 10.1016/j.jclinepi.2010.07.015 21208779

[15] Chen ST Lu KH Sun HL Chang WT Lue KH Chou MC. Randomized placebo-controlled trial comparing montelukast and cetirizine for treating perennial allergic rhinitis in children aged 2-6 yr. *Pediatr Allergy Immunol.* (2006) 17:49–54. 10.1111/j.1399-3038.2005.00351.x 16426255

[16] Delgado LF Pferferman A Sole D Naspitz CK. Evaluation of the potential cardiotoxicity of the antihistamines terfenadine, astemizole, loratadine, and cetirizine in atopic children. *Ann Allergy Asthma Immunol.* (1998) 80:333–7. 10.1016/S1081-1206(10)62979-1 9564984

[17] Hsieh JC Lue KH Lai DS Sun HL Lin YH. A comparison of cetirizine and montelukast for treating childhood perennial allergic rhinitis. *Pediatr Asthma Allergy Immunol.* (2004) 17:59–69. 10.1089/088318704322994958

[18] Jobst S van den Wijngaart W Schubert A van de Venne H. Assessment of the efficacy and safety of three dose levels of cetirizine given once daily in children with perennial allergic rhinitis. *Allergy.* (1994) 49:598–604. 10.1111/j.1398-9995.1994.tb00125.x 7653736

[19] Lai DS Lue KH Hsieh JC Lin KL Lee HS. The comparison of the efficacy and safety of cetirizine, oxatomide, ketotifen, and a placebo for the treatment of childhood perennial allergic rhinitis. *Ann Allergy Asthma Immunol.* (2002) 89:589–98. 10.1016/S1081-1206(10)62107-2 12487225

[20] Lee CF Sun HL Lu KH Ku MS Lue KH. The comparison of cetirizine, levocetirizine and placebo for the treatment of childhood perennial allergic rhinitis. *Pediatr Allergy Immunol.* (2009) 20:493–9. 10.1111/j.1399-3038.2008.00816.x 19175892

[21] Ng KH Chong D Wong CK Ong HT Lee CY Lee BW Central nervous system side effects of first- and second-generation antihistamines in school children with perennial allergic rhinitis: a randomized, double-blind, placebo-controlled comparative study. *Pediatrics.* (2004) 113:e116–21. 10.1542/peds.113.2.e116 14754980

[22] Sienra-Monge JJ Gazca-Aguilar A Del Rio-Navarro B. Double-blind comparison of cetirizine and loratadine in children ages 2 to 6 years with perennial allergic rhinitis. *Am J Ther.* (1999) 6:149–55. 10.1097/00045391-199905000-00005 10423657

[23] Allegra L Paupe J Wieseman HG Baelde Y. Cetirizine for seasonal allergic rhinitis in children aged 2-6 years. A double-blind comparison with placebo. *Pediatr Allergy Immunol.* (1993) 4:157–61. 10.1111/j.1399-3038.1993.tb00085.x 8220804

[24] Nayak AS Berger WE LaForce CF Urdaneta ER Patel MK Franklin KB Randomized, placebo-controlled study of cetirizine and loratadine in children with seasonal allergic rhinitis. *Allergy Asthma Proc.* (2017) 38:222–30. 10.2500/aap.2017.38.4050 28441993

[25] Pearlman DS Lumry WR Winder JA Noonan MJ. Once-daily cetirizine effective in the treatment of seasonal allergic rhinitis in children aged 6 to 11 years: a randomized, double-blind, placebo-controlled study. *Clin Pediatr (Phila).* (1997) 36:209–15. 10.1177/000992289703600405 9114992

[26] Tinkelman DG Kemp J Mitchell DQ Galant SP. Treatment of seasonal allergic rhinitis in children with cetirizine or chlorpheniramine: a multicenter study. *Pediatr Asthma Allergy Immunol.* (1996) 10:9–17. 10.1089/pai.1996.10.9

[27] Winder JA Noonan MJ Lumry WR Pearlman DS. Absence of QT c prolongation with cetirizine in children aged 6 to 11 years. *Pediatr Asthma Allergy Immunol.* (1996) 10:181–90. 10.1089/pai.1996.10.181

[28] Segal AT Meltzer EO Lockey RF Prenner BM Mitchell DQ Tinkelman DG Once-daily cetirizine is safe and effective for children with allergic rhinitis with and without intermittent asthma. *Pediatr Asthma Allergy Immunol.* (2003) 16:265–74. 10.1089/088318703322751318 15264995

[29] Baelde Y. Cetirizine in children with chronic allergic rhinitis: a multicentre double-blind study of two doses of cetirizine and placebo. *Drug Invest.* (1992) 4:466–72. 10.1007/BF03259210

[30] Simons FE Silas P Portnoy JM Catuogno J Chapman D Olufade AO. Safety of cetirizine in infants 6 to 11 months of age: a randomized, double-blind, placebo-controlled study. *J Allergy Clin Immunol.* (2003) 111:1244–8. 10.1067/mai.2003.1496 12789224

[31] Diepgen TL Early Treatment of the Atopic Child Study Group. Long-term treatment with cetirizine of infants with atopic dermatitis: a multi-country, double-blind, randomized, placebo-controlled trial (the ETAC trial) over 18 months. *Pediatr Allergy Immunol.* (2002) 13:278–86. 10.1034/j.1399-3038.2002.01047.x 12390444

[32] Simons FE. Prospective, long-term safety evaluation of the H1-receptor antagonist cetirizine in very young children with atopic dermatitis. ETAC study group. Early treatment of the atopic child. *J Allergy Clin Immunol.* (1999) 104(2 Pt 1):433–40. 10.1016/S0091-6749(99)70389-1 10452767

[33] Wahn UJ. Allergic factors associated with the development of asthma and the influence of cetirizine in a double-blind, randomised, placebo-controlled trial: first results of ETA§. *Pediatr Allergy Immunol.* (1998) 9:116–24. 10.1111/j.1399-3038.1998.tb00356.x 9814724

[34] Warner JO Etac Study Group. Early Treatment of the Atopic Child. A double-blinded, randomized, placebo-controlled trial of cetirizine in preventing the onset of asthma in children with atopic dermatitis: 18 months’ treatment and 18 months’ posttreatment follow-up. *J Allergy Clin Immunol.* (2001) 108:929–37. 10.1067/mai.2001.120015 11742270

[35] Masi M Candiani R van de Venne H. A placebo-controlled trial of cetirizine in seasonal allergic rhino-conjunctivitis in children aged 6 to 12 years. *Pediatr Allergy Immunol.* (1993) 4(Suppl. 4):47–52. 10.1111/j.1399-3038.1993.tb00339.x 8353660

[36] Ciprandi G Tosca M Passalacqua G Canonica GW. Long-term cetirizine treatment reduces allergic symptoms and drug prescriptions in children with mite allergy. *Ann Allergy Asthma Immunol.* (2001) 87:222–6. 10.1016/S1081-1206(10)62230-2 11570619

[37] Yanai K Rogala B Chugh K Paraskakis E Pampura AN Boev R. Safety considerations in the management of allergic diseases: focus on antihistamines. *Curr Med Res Opin.* (2012) 28:623–42. 10.1185/03007995.2012.672405 22455874

[38] Dykewicz MS Wallace DV Amrol DJ Baroody FM Bernstein JA Craig TJ Rhinitis 2020: a practice parameter update. *J Allergy Clin Immunol.* (2020) 146:721–67. 10.1016/j.jaci.2020.07.007 32707227

[39] Curran MP Scott LJ Perry CM. Cetirizine: a review of its use in allergic disorders. *Drugs.* (2004) 64:523–61. 10.2165/00003495-200464050-00008 14977391

[40] Caffarelli C Paravati F El Hachem M Duse M Bergamini M Simeone G Management of chronic urticaria in children: a clinical guideline. *Ital J Pediatr.* (2019) 45:101. 10.1186/s13052-019-0695-x 31416456 PMC6694633

[41] Gillard M Christophe B Wels B Peck M Massingham R Chatelain P. H1 antagonists: receptor affinity versus selectivity. *Inflamm Res.* (2003) 52(Suppl 1):S49–50. 10.1007/s000110300050 12755407

[42] Miligkos M Dakoutrou M Statha E Theochari NA Mavroeidi IA Pankozidou I Newer-generation antihistamines and the risk of adverse events in children: a systematic review. *Pediatr Allergy Immunol.* (2021) 32:1533–58. 10.1111/pai.13522 33894089

[43] Stevenson J Cornah D Evrard P Vanderheyden V Billard C Bax M Long-term evaluation of the impact of the h1-receptor antagonist cetirizine on the behavioral, cognitive, and psychomotor development of very young children with atopic dermatitis. *Pediatr Res.* (2002) 52:251–7. 10.1203/00006450-200208000-00018 12149503

[44] van Ruitenbeek P Vermeeren A Riedel WJ. Histamine H1 receptor antagonist cetirizine impairs working memory processing speed, but not episodic memory. *Br J Pharmacol.* (2010) 161:456–66. 10.1111/j.1476-5381.2010.00907.x 20735428 PMC2989595

[45] van Ruitenbeek P Vermeeren A Riedel W. Histamine H1-receptor blockade in humans affects psychomotor performance but not memory. *J Psychopharmacol.* (2008) 22:663–72. 10.1177/0269881107081526 18208925

[46] Hindmarch I Johnson S Meadows R Kirkpatrick T Shamsi Z. The acute and sub-chronic effects of levocetirizine, cetirizine, loratadine, promethazine and placebo on cognitive function, psychomotor performance, and weal and flare. *Curr Med Res Opin.* (2001) 17:241–55. 10.1185/0300799019117011 11922397

[47] Ali Z Ismail M Khan F Sajid H. Association of H1-antihistamines with torsade de pointes: a pharmacovigilance study of the food and drug administration adverse event reporting system. *Expert Opin Drug Saf.* (2021) 20:101–7. 10.1080/14740338.2021.1846717 33141610

[48] Poluzzi E Raschi E Godman B Koci A Moretti U Kalaba M Pro-arrhythmic potential of oral antihistamines (H1): combining adverse event reports with drug utilization data across Europe. *PLoS One.* (2015) 10:e0119551. 10.1371/journal.pone.0119551 25785934 PMC4364720

[49] Phan H Moeller ML Nahata MC. Treatment of allergic rhinitis in infants and children: efficacy and safety of second-generation antihistamines and the leukotriene receptor antagonist montelukast. *Drugs.* (2009) 69:2541–76. 10.2165/9884960-000000000-00000 19943707

[50] Cataldi M Maurer M Taglialatela M Church MK. Cardiac safety of second-generation H1 -antihistamines when updosed in chronic spontaneous urticaria. *Clin Exp Allergy.* (2019) 49:1615–23. 10.1111/cea.13500 31519068

[51] Powell RJ Du Toit GL Siddique N Leech SC Dixon TA Clark AT BSACI guidelines for the management of chronic urticaria and angio-oedema. *Clin Exp Allergy.* (2007) 37:631–50. 10.1111/j.1365-2222.2007.02678.x 17456211

[52] Zuberbier T Aberer W Asero R Abdul Latiff AH Baker D Ballmer-Weber B The EAACI/GA LEN/EDF/WAO guideline for the definition, classification, diagnosis and management of urticaria. *Allergy.* (2018) 73:1393–414. 29336054 10.1111/all.13397

[53] Sanchez-Borges M Caballero-Fonseca F Capriles-Hulett A. Treatment of recalcitrant chronic urticaria with nonsedating antihistamines: is there evidence for updosing? *J Investig Allergol Clin Immunol.* (2013) 23:141–144;quiz142receding145. 23967751

[54] Asero R. Chronic unremitting urticaria: is the use of antihistamines above the licensed dose effective? A preliminary study of cetirizine at licensed and above-licensed doses. *Clin Exp Dermatol.* (2007) 32:34–8. 10.1111/j.1365-2230.2006.02278.x 17042777

[55] Blaiss MS Bernstein JA Kessler A Pines JM Camargo CA Jr. Fulgham P The role of cetirizine in the changing landscape of IV antihistamines: a narrative review. *Adv Ther.* (2022) 39:178–92. 10.1007/s12325-021-01999-x 34862952 PMC8643118

[56] Thangam EB Jemima EA Singh H Baig MS Khan M Mathias CB The role of histamine and histamine receptors in mast cell-mediated allergy and inflammation: the hunt for new therapeutic targets. *Front Immunol.* (2018) 9:1873. 10.3389/fimmu.2018.01873 30150993 PMC6099187

[57] Church MK. Allergy, histamine and antihistamines. *Handb Exp Pharmacol.* (2017) 241:321–31. 10.1007/164_2016_85 28101683

[58] Sterne JA Sutton AJ Ioannidis JP Terrin N Jones DR Lau J Recommendations for examining and interpreting funnel plot asymmetry in meta-analyses of randomised controlled trials. *BMJ.* (2011) 343:d4002. 10.1136/bmj.d4002 21784880

