# Supplementary material for: Cetirizine for the treatment of allergic diseases in children: A systematic review and meta-analysis
Source: Front Pediatr. 2022 Aug 25;10:940213. doi: 10.3389/fped.2022.940213 (PMC9452751; doi:10.3389/fped.2022.940213)
Supplement: Supplementary file 1 [file Data_Sheet_1.pdf]

Supplementary Files

Supplementary File 1 PRISMA Checklist

Section and Topic	Item #	Checklist item	Location where item is reported
**TITLE**			
Title	1	Identify the report as a systematic review.	Page 1
**ABSTRACT**			
Abstract	2	See the PRISMA 2020 for Abstracts checklist.	Page 1-2
**INTRODUCTION**			
Rationale	3	Describe the rationale for the review in the context of existing knowledge.	Page 2
Objectives	4	Provide an explicit statement of the objective(s) or question(s) the review addresses.	Page 2
**METHODS**			
Eligibility criteria	5	Specify the inclusion and exclusion criteria for the review and how studies were grouped for the syntheses.	Page 3
Information sources	6	Specify all databases, registers, websites, organisations, reference lists and other sources searched or consulted to identify studies. Specify the date when each source was last searched or consulted.	Page 3

Section and Topic	Item #	Checklist item	Location where item is reported
Search strategy	7	Present the full search strategies for all databases, registers and websites, including any filters and limits used.	Page 3 and Supplementary File 2
Selection process	8	Specify the methods used to decide whether a study met the inclusion criteria of the review, including how many reviewers screened each record and each report retrieved, whether they worked independently, and if applicable, details of automation tools used in the process.	Page 3
Data collection process	9	Specify the methods used to collect data from reports, including how many reviewers collected data from each report, whether they worked independently, any processes for obtaining or confirming data from study investigators, and if applicable, details of automation tools used in the process.	Page 3
Data items	10a	List and define all outcomes for which data were sought. Specify whether all results that were compatible with each outcome domain in each study were sought (e.g. for all measures, time points, analyses), and if not, the methods used to decide which results to collect.	Page 3
	10b	List and define all other variables for which data were sought (e.g. participant and intervention characteristics, funding sources). Describe any assumptions made about any missing or unclear information.	Page 3
Study risk of bias assessment	11	Specify the methods used to assess risk of bias in the included studies, including details of the tool(s) used, how many reviewers assessed each study and whether they worked independently,	Page 3

Section and Topic	Item #	Checklist item	Location where item is reported
		and if applicable, details of automation tools used in the process.	
Effect measures	12	Specify for each outcome the effect measure(s) (e.g. risk ratio, mean difference) used in the synthesis or presentation of results.	Page 3
Synthesis methods	13a	Describe the processes used to decide which studies were eligible for each synthesis (e.g. tabulating the study intervention characteristics and comparing against the planned groups for each synthesis (item #5)).	Page 3-4
	13b	Describe any methods required to prepare the data for presentation or synthesis, such as handling of missing summary statistics, or data conversions.	Page 4
	13c	Describe any methods used to tabulate or visually display results of individual studies and syntheses.	Page 4
	13d	Describe any methods used to synthesize results and provide a rationale for the choice(s). If meta-analysis was performed, describe the model(s), method(s) to identify the presence and extent of statistical heterogeneity, and software package(s) used.	Page 4
	13e	Describe any methods used to explore possible causes of heterogeneity among study results (e.g. subgroup analysis, meta-regression).	Page 4

Section and Topic	Item #	Checklist item	Location where item is reported
	13f	Describe any sensitivity analyses conducted to assess robustness of the synthesized results.	
Reporting bias assessment	14	Describe any methods used to assess risk of bias due to missing results in a synthesis (arising from reporting biases).	Page 4
Certainty assessment	15	Describe any methods used to assess certainty (or confidence) in the body of evidence for an outcome.	Page 3
**RESULTS**			
Study selection	16a	Describe the results of the search and selection process, from the number of records identified in the search to the number of studies included in the review, ideally using a flow diagram.	Page 4 and Figure 1
	16b	Cite studies that might appear to meet the inclusion criteria, but which were excluded, and explain why they were excluded.	Page 4 and Figure 1
Study characteristics	17	Cite each included study and present its characteristics.	Page 4 and Table 1
Risk of bias in studies	18	Present assessments of risk of bias for each included study.	Page 4, Figure 2, Figure 3 and Supplementary File 3
Results of individual studies	19	For all outcomes, present, for each study: (a) summary statistics for each group (where appropriate)	Page 4 and Table 1

Section and Topic	Item #	Checklist item	Location where item is reported
		and (b) an effect estimates and its precision (e.g. confidence/credible interval), ideally using structured tables or plots.	
Results of syntheses	20a	For each synthesis, briefly summarise the characteristics and risk of bias among contributing studies.	Page 5-7, Figure 2, and Figure 3
	20b	Present results of all statistical syntheses conducted. If meta-analysis was done, present for each the summary estimate and its precision (e.g. confidence/credible interval) and measures of statistical heterogeneity. If comparing groups, describe the direction of the effect.	Page 5-7, Figure 4, Supplementary File 4, and Supplementary File 5
	20c	Present results of all investigations of possible causes of heterogeneity among study results.	Page 5-7
	20d	Present results of all sensitivity analyses conducted to assess the robustness of the synthesized results.	Page 5-7
Reporting biases	21	Present assessments of risk of bias due to missing results (arising from reporting biases) for each synthesis assessed.	NA*
Certainty of evidence	22	Present assessments of certainty (or confidence) in the body of evidence for each outcome assessed.	Page 5-7 and Supplementary File 6

Section and Topic	Item #	Checklist item	Location where item is reported
**DISCUSSION**			
Discussion	23a	Provide a general interpretation of the results in the context of other evidence.	Page 7
	23b	Discuss any limitations of the evidence included in the review.	Page 8
	23c	Discuss any limitations of the review processes used.	Page 8
	23d	Discuss implications of the results for practice, policy, and future research.	Page 8
**OTHER INFORMATION**			
Registration and protocol	24a	Provide registration information for the review, including register name and registration number, or state that the review was not registered.	Page 1 and Page 3
	24b	Indicate where the review protocol can be accessed, or state that a protocol was not prepared.	Page 1 and Page 3
	24c	Describe and explain any amendments to information provided at registration or in the protocol.	Page 1 and Page 3
Support	25	Describe sources of financial or non-financial support for the review, and the role of the funders or sponsors in the review.	Page 9
Competing interests	26	Declare any competing interests of review authors.	Page 9
Availability of data, code and other materials	27	Report which of the following are publicly available and where they can be found: template data collection forms; data extracted from included studies; data used for all analyses; analytic code; any	Page 14

Section and Topic	Item #	Checklist item	Location where item is reported
		other materials used in the review.	

* Publication bias was conducted using Revman 5.4 software, but we did not show the results in the manuscript because of insufficient studies for individual outcome.

Supplementary File 2 Search strategies

Pubmed

#1 "cetirizine"[MeSH Terms] OR "cetirizine"[All Fields]

#2 randomized controlled trial [pt] OR controlled clinical trial [pt] OR randomized [tiab] OR placebo [tiab] OR clinical trials as topic [mesh: noexp] OR randomly [tiab] OR trial [ti]

#3 infant* OR neonat* OR newborn OR teen* OR preschool* OR school* OR adolescen* OR toddler* OR pubert* OR minor* OR prematur* OR juvenile OR pediatric* OR paediatric* OR child* OR girl OR boy OR baby OR kid

#4 animals [mh] NOT humans [mh]

#5 (#1 AND #2 AND #3) NOT #4

Embase

#1 'cetirizine'/exp OR cetirizine

#2 random*:ab,ti OR placebo*:de,ab,ti OR ((double NEXT/1 blind*):ab,ti)

#3 [newborn]/lim OR [infant]/lim OR [child]/lim OR [preschool]/lim OR [school]/lim OR [adolescent]/lim

#4 [humans]/lim

#5 #1 AND #2 AND #3 AND #4

the Cochrane Central Register of Controlled Trials (CENTRAL)

#1 cetirizine

#2 MeSH descriptor: [Child] explode all trees OR MeSH descriptor: [Infant] explode all trees OR MeSH descriptor: [Adolescent] explode all trees

#3 #1 AND #2

Register Platform

Clinicaltrials.gov: cetirizine | Studies with results | Child (birth-17)

the International Clinical Trials Registry Platform: cetirizine, with results only

the European Union Clinical Trials Register: cetirizine

Supplementary File 3 Reasons of Risk of Bias Assessment of Included Trials

Included trials	Selection bias (Randomization)	Selection bias (Allocation concealment)	Performance bias	Detection bias	Attrition bias	Reporting bias	Other bias
Chen 2006	NA	NA	double-blinded	NA	No missing data	All predesigned outcomes were reported.	The authors declared the conflict of interest.
Delgado 1998	NA	NA	NA	NA	No missing data	All predesigned outcomes were reported.	No declared conflict of interest
Hsieh 2004	computer-generated randomization code	NA	double-blinded	NA	Missing data did not affect the consistency between groups	All predesigned outcomes were reported.	No declared conflict of interest
Jobst S 1994	computer-generated block randomization	NA	double-blinded	NA	Missing data did not affect the consistency between groups	All predesigned outcomes were reported.	No declared conflict of interest

Lai 2002	computer-generated randomization code	NA	double-blinded	NA	Missing data did not affect the consistency between groups	All predesigned outcomes were reported.	No declared conflict of interest
Lee 2009	NA	NA	double-blinded	NA	Missing data did not affect the consistency between groups	All predesigned outcomes were reported.	No declared conflict of interest
Ng 2004	a table of random numbers	medications were prepared by the pharmacist	double-blinded	the investigators were blinded	No missing data	All predesigned outcomes were reported.	No declared conflict of interest
Sienra-monge 1999	NA	NA	double-blinded	NA	No missing data. Except for weight, the consistency between groups were confirmed*.	All predesigned outcomes were reported.	No declared conflict of interest

Allegra L 1993	a computer-generated block randomization list	NA	double-blinded	NA	No missing data	All predesigned outcomes were reported.	No declared conflict of interest
Nayak 2017	NA	NA	triple (participant, care provider, investigator)	triple (participant, care provider, investigator)	Missing data did not affect the consistency between groups	All predesigned outcomes were reported.	Part of authors were employees of the company, but they did not declare the conflict of interest.
Pearlman 1997	NA	NA	double-blinded	NA	Missing data did not possibly affect the consistency between groups, except for	All predesigned outcomes were reported.	No declared conflict of interest

					gender and race*.		
Tinkelman 1996	NA	the study nurse conducted drug assignment and dispensing in a blind fashion	single-blinded	NA	Missing data did not affect the consistency between groups	All predesigned outcomes were reported.	No declared conflict of interest
Winder 1996	NA	NA	double-blinded	NA	Missing data did not possibly affect the consistency between groups, except for gender and race*.	All predesigned outcomes were reported.	No declared conflict of interest
Segal 2003	NA	NA	double-blinded	NA	Missing data did not affect the consistency between groups	All predesigned outcomes were reported.	No declared conflict of interest

Baelde 1992	computer-generated randomization	NA	double-blinded	NA	Missing data did not affect the consistency between groups	All predesigned outcomes were reported.	No declared conflict of interest
Simons 2003	NA	NA	double-blinded	NA	Missing data did not affect the consistency between groups	All predesigned outcomes were reported.	No declared conflict of interest
Diepgen 2002	a randomization list by blocks of 2, created using a computer software	treatments assigned to treatment codes independently by the clinical research organization	double-blinded	All data were calculated and reviewed before database lock by an independent Data Review Committee including Scientific Advisory Board members	Missing data did not affect the consistency between groups	All predesigned outcomes were reported.	No declared conflict of interest
Simons 1999	a randomization	treatments assigned to	double-blinded	All data were calculated and	Missing data did not affect the	All predesigned outcomes were reported.	No declared conflict of interest

	list by blocks of 2, created using a computer software	treatment codes independently by the clinical research organization		reviewed before database lock by an independent Data Review Committee including Scientific Advisory Board members	consistency between groups		
Wahn 1998	a randomization list by blocks of 2, created using a computer software	treatments assigned to treatment codes independently by the clinical research organization	double-blinded	All data were calculated and reviewed before database lock by an independent Data Review Committee including Scientific Advisory Board members	Missing data did not affect the consistency between groups	All predesigned outcomes were reported.	No declared conflict of interest
Warner 2001	a randomization list by blocks	treatments assigned to treatment codes	double-blinded	All data were calculated and reviewed before	Missing data did not possibly affect the	All predesigned outcomes were reported.	No declared conflict of interest

	of 2, created using a computer software	independently by the clinical research organization		database lock by an independent Data Review Committee including Scientific Advisory Board members	consistency between groups, except for egg sensitization. Multivariate analysis adjusted the bias from egg sensitization.		
Masi 1993	NA	NA	double-blinded	NA	Missing data did not affect the consistency between groups	All predesigned outcomes were reported.	No declared conflict of interest
Ciprandi 2001	randomly coded	NA	double-blinded	NA	No missing data	All predesigned outcomes were reported.	No declared conflict of interest

Note:

U: unclear risk of bias; L: low risk of bias; H: high risk of bias.

The yellow background represents unclear risk of bias; The green background represents low risk of bias; The red background represents high risk of bias;

Supplementary File 4 Comparison of Cetirizine with Placebo or Other Drugs in Efficacy Evaluation

Supplementary File 4.1 Serum Total IgE

	CTZ group			Control group				Mean Difference
**Study or Subgroup**	**Mean**	**SD**	**Total**	**Mean**	**SD**	**Total**	**Weight**	**IV, Fixed, 95% CI**
**2.1.1 CTZ vs placebo**								
Chen 2006	−71.5	196.24	20	−10.6	41.93	20	39.2%	−60.90 [−148.85, 27.05]
Hsieh 2004	−33.65	209.15	20	71	174.85	20	21.2%	−104.65 [−224.12, 14.82]
Lai 2002	−27.95	214.69	19	90	188.94	16	16.9%	−117.95 [−251.70, 15.80]
Lee 2009	−33.56	204.33	26	71	211.68	24	22.7%	−104.56 [−220.06, 10.94]
**Subtotal (95% CI)**			**85**			**80**	**100.0%**	**−89.75 [−144.78, −34.72]**
Heterogeneity: $Chi^2 = 0.71$, df = 3 (P = 0.87); $I^2 = 0\%$								
Test for overall effect: Z = 3.20 (P = 0.001)								
**2.1.2 CTZ vs other AHs**								
Lai 2002	−27.95	214.69	19	154.78	264.13	16	48.6%	−182.73 [−344.19, −21.27]
Lee 2009	−33.56	204.23	26	7.72	339.52	24	51.4%	−41.28 [−198.17, 115.61]
**Subtotal (95% CI)**			**45**			**40**	**100.0%**	**−109.97 [−222.49, 2.54]**
Heterogeneity: $Chi^2 = 1.52$, df = 1 (P = 0.22); $I^2 = 34\%$								
Test for overall effect: Z = 1.92 (P = 0.06)								
**2.1.3 CTZ vs montelukast**								
Chen 2006	−71.5	196.24	20	−37.15	123.59	20	75.2%	−34.35 [−135.99, 67.29]
Hsieh 2004	−33.65	209.15	20	20.18	345.11	20	24.8%	−53.83 [−230.69, 123.03]
**Subtotal (95% CI)**			**40**			**40**	**100.0%**	**−39.19 [−127.31, 48.94]**
Heterogeneity: $Chi^2 = 0.04$, df = 1 (P = 0.85); $I^2 = 0\%$								
Test for overall effect: Z = 0.87 (P = 0.38)								

Mean Difference
IV, Fixed, 95% CI

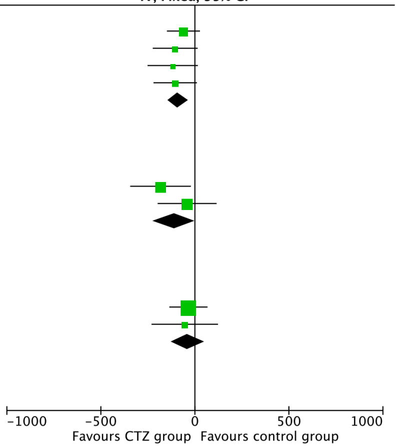

Test for subgroup differences: $Chi^2 = 1.22$, df = 2 (P = 0.54), $I^2 = 0\%$

Supplementary File 4.2 ECP

	CTZ group			Control group				Mean Difference
**Study or Subgroup**	**Mean**	**SD**	**Total**	**Mean**	**SD**	**Total**	**Weight**	**IV, Random, 95% CI**
**3.1.1 CTZ vs placebo**								
Chen 2006	−2.35	11.03	20	0.15	3.61	20	5.5%	−2.50 [−7.59, 2.59]
Hsieh 2004	−2.68	3.12	20	1.11	2.93	20	40.4%	−3.79 [−5.67, −1.91]
Lai 2002	−2.5	3.2	19	1.52	2.64	16	38.0%	−4.02 [−5.95, −2.09]
Lee 2009	−2.68	7.02	26	1.11	3.09	24	16.1%	−3.79 [−6.76, −0.82]
**Subtotal (95% CI)**			**85**			**80**	**100.0%**	**−3.81 [−5.00, −2.61]**
Heterogeneity: $Tau^2 = 0.00$; $Chi^2 = 0.30$, df = 3 (P = 0.96); $I^2 = 0\%$								
Test for overall effect: Z = 6.26 (P < 0.00001)								
**3.1.2 CTZ vs other AHs**								
Lai 2002	−2.5	3.2	19	4.45	7.63	16	52.7%	−6.95 [−10.96, −2.94]
Lee 2009	−2.68	7.02	26	−13.56	21.56	24	47.3%	10.88 [1.84, 19.92]
**Subtotal (95% CI)**			**45**			**40**	**100.0%**	**1.49 [−15.96, 18.93]**
Heterogeneity: $Tau^2 = 146.23$; $Chi^2 = 12.50$, df = 1 (P = 0.0004); $I^2 = 92\%$								
Test for overall effect: Z = 0.17 (P = 0.87)								
**3.1.3 CTZ vs montelukast**								
Chen 2006	−2.35	11.03	20	−1.75	24.4	20	9.4%	−0.60 [−12.34, 11.14]
Hsieh 2004	−2.68	3.12	20	−1.91	8.06	20	90.6%	−0.77 [−4.56, 3.02]
**Subtotal (95% CI)**			**40**			**40**	**100.0%**	**−0.75 [−4.36, 2.85]**
Heterogeneity: $Tau^2 = 0.00$; $Chi^2 = 0.00$, df = 1 (P = 0.98); $I^2 = 0\%$								
Test for overall effect: Z = 0.41 (P = 0.68)								

Mean Difference
IV, Random, 95% CI

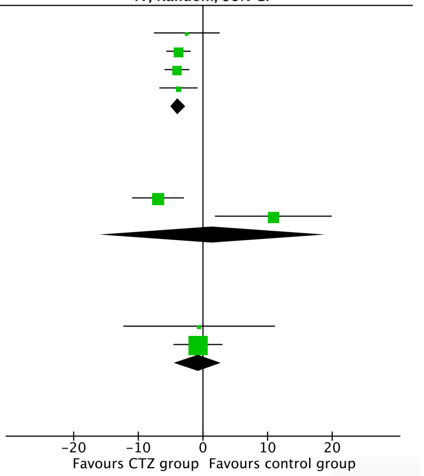

Test for subgroup differences: $Chi^2 = 2.80$, df = 2 (P = 0.25), $I^2 = 28.5\%$

Supplementary File 4.3 Blood Eosinophil Counts

Study or Subgroup	CTZ group Mean	SD	Total	Control group Mean	SD	Total	Weight	Mean Difference IV, Fixed, 95% CI
**4.1.1 CTZ vs placebo**								
Chen 2006	−60.65	384.19	20	2.95	51.98	20	14.3%	−63.60 [−233.51, 106.31]
Hsieh 2004	−118.15	180.56	20	56.5	170.74	20	34.7%	−174.65 [−283.56, −65.74]
Lai 2002	−118.05	181.21	19	67.5	179.95	16	28.6%	−185.55 [−305.61, −65.49]
Lee 2009	−118.15	272.44	26	56.5	214.14	24	22.5%	−174.65 [−309.95, −39.35]
**Subtotal (95% CI)**			**85**			**80**	**100.0%**	**−161.93 [−226.09, −97.77]**
Heterogeneity: $Chi^2 = 1.52$, df = 3 (P = 0.68); $I^2 = 0\%$								
Test for overall effect: Z = 4.95 (P < 0.00001)								
**4.1.2 CTZ vs other AHs**								
Lai 2002	−118.05	181.21	19	23.25	101.76	16	69.0%	−141.30 [−236.83, −45.77]
Lee 2009	−118.15	272.44	26	−45.38	241.41	24	31.0%	−72.77 [−215.23, 69.69]
**Subtotal (95% CI)**			**45**			**40**	**100.0%**	**−120.04 [−199.38, −40.70]**
Heterogeneity: $Chi^2 = 0.61$, df = 1 (P = 0.43); $I^2 = 0\%$								
Test for overall effect: Z = 2.97 (P = 0.003)								
**4.1.3 CTZ vs montelukast**								
Chen 2006	−60.65	384.19	20	−67.5	291.04	20	28.1%	6.85 [−204.38, 218.08]
Hsieh 2004	−118.15	180.56	20	−45.38	241.41	20	71.9%	−72.77 [−204.89, 59.35]
**Subtotal (95% CI)**			**40**			**40**	**100.0%**	**−50.38 [−162.39, 61.63]**
Heterogeneity: $Chi^2 = 0.39$, df = 1 (P = 0.53); $I^2 = 0\%$								
Test for overall effect: Z = 0.88 (P = 0.38)								

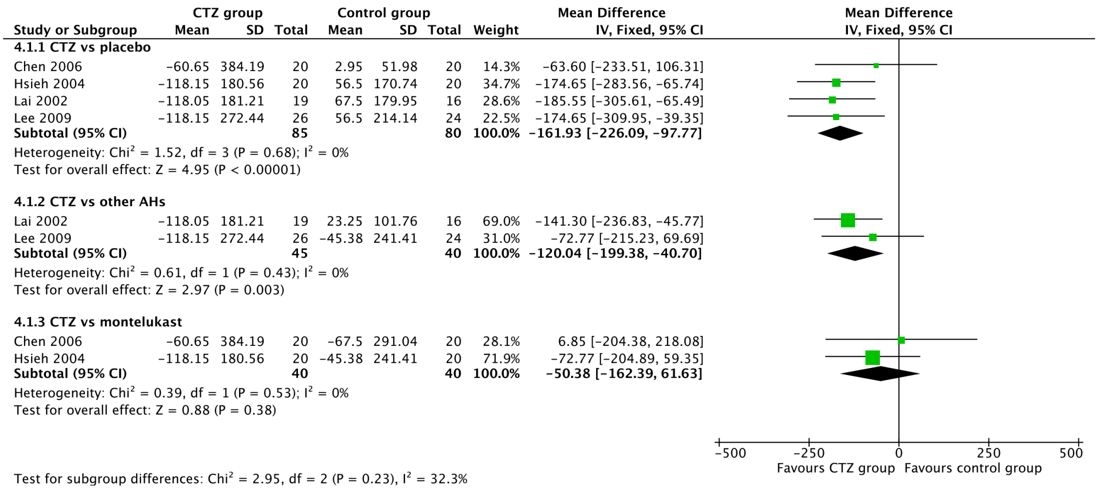

Test for subgroup differences: $Chi^2 = 2.95$, df = 2 (P = 0.23), $I^2 = 32.3\%$

Supplementary File 4.4 PRQLQ

Study or Subgroup	CTZ group Mean	SD	Total	Control group Mean	SD	Total	Weight	Mean Difference IV, Random, 95% CI
**5.1.1 CTZ vs placebo**								
Chen 2006	−31.15	23.36	20	−3.85	5.56	20	12.8%	−27.30 [−37.82, −16.78]
Lai 2002	−21.07	12.04	19	4.73	11.47	16	23.3%	−25.80 [−33.60, −18.00]
Lee 2009	−19.73	11.04	26	1.63	5.13	24	63.9%	−21.36 [−26.07, −16.65]
**Subtotal (95% CI)**			**65**			**60**	**100.0%**	**−23.16 [−26.92, −19.39]**
Heterogeneity: $Tau^2 = 0.00$; $Chi^2 = 1.59$, df = 2 (P = 0.45); $I^2 = 0\%$								
Test for overall effect: Z = 12.05 (P < 0.00001)								
**5.1.2 CTZ vs other AHs**								
Lai 2002	−21.07	12.04	19	−14.06	17.37	16	46.1%	−7.01 [−17.10, 3.08]
Lee 2009	−19.73	11.04	26	−24.09	16.82	24	53.9%	4.36 [−3.60, 12.32]
**Subtotal (95% CI)**			**45**			**40**	**100.0%**	**−0.88 [−11.99, 10.22]**
Heterogeneity: $Tau^2 = 43.16$; $Chi^2 = 3.01$, df = 1 (P = 0.08); $I^2 = 67\%$								
Test for overall effect: Z = 0.16 (P = 0.88)								
**5.1.3 CTZ vs montelukast**								
Chen 2006	−31.15	23.36	20	−19.15	20.71	20	100.0%	−12.00 [−25.68, 1.68]
**Subtotal (95% CI)**			**20**			**20**	**100.0%**	**−12.00 [−25.68, 1.68]**
Heterogeneity: Not applicable								
Test for overall effect: Z = 1.72 (P = 0.09)								

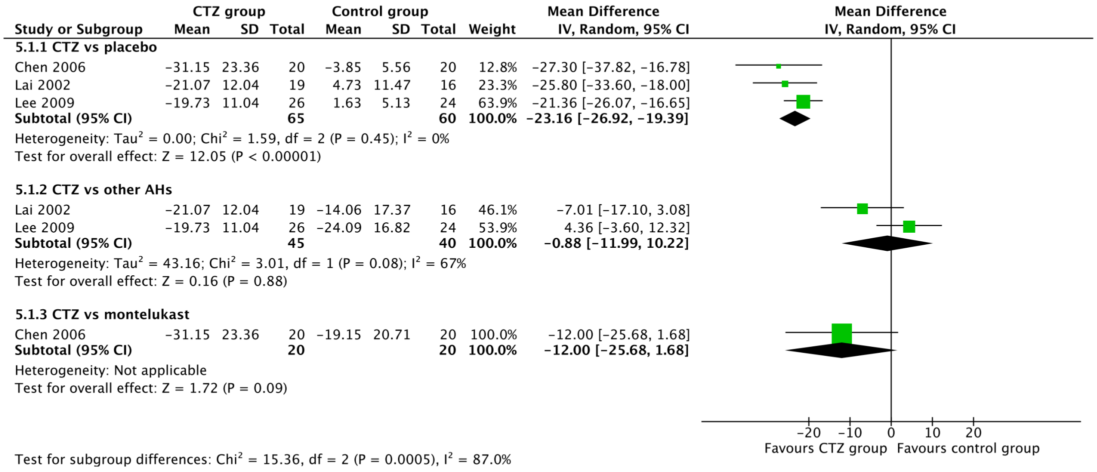

Test for subgroup differences: $Chi^2 = 15.36$, df = 2 (P = 0.0005), $I^2 = 87.0\%$

Supplementary File 5 Comparison of Cetirizine with Placebo or Other Drugs in Safety Evaluation

Supplementary File 5.1 Overall AEs

Study or Subgroup	CTZ group Events	CTZ group Total	Control group Events	Control group Total	Weight	Risk Ratio M-H, Fixed, 95% CI
Allegra L 1993	13	54	11	53	10.9%	1.16 [0.57, 2.35]
Baelde 1992	11	28	6	18	7.2%	1.18 [0.53, 2.62]
Hsieh 2004	1	20	2	20	2.0%	0.50 [0.05, 5.08]
Jobst S 1994	50	244	15	83	22.1%	1.13 [0.67, 1.91]
Masi 1993	14	63	14	61	14.0%	0.97 [0.50, 1.86]
Segal 2003	27	81	18	83	17.5%	1.54 [0.92, 2.56]
Simons 2003	19	42	27	43	26.3%	0.72 [0.48, 1.08]
**Total (95% CI)**		**532**		**361**	**100.0%**	**1.07 [0.85, 1.34]**
Total events	135		93			

Heterogeneity: Chi^2 = 6.23, df = 6 (P = 0.40); I^2 = 4%
Test for overall effect: Z = 0.55 (P = 0.58)

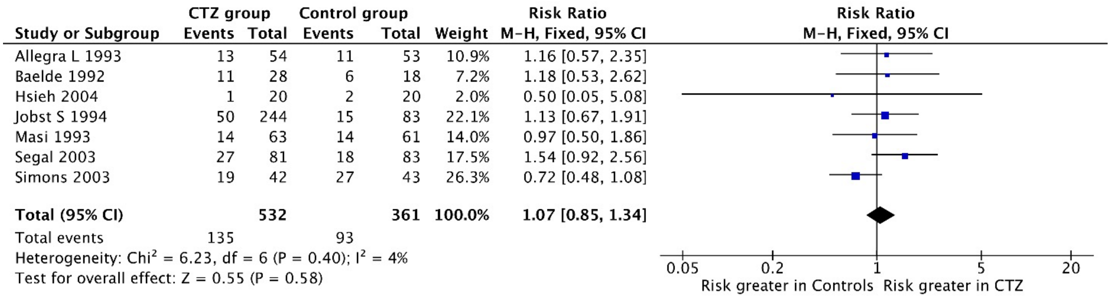

Supplementary File 5.2 Somnolence

Study or Subgroup	CTZ group Events	CTZ group Total	Control group Events	Control group Total	Weight	Risk Ratio M-H, Fixed, 95% CI
**6.1.1 CTZ vs placebo**						
Allegra L 1993	3	18	0	15	2.9%	5.89 [0.33, 105.81]
Chen 2006	2	20	0	20	2.6%	5.00 [0.26, 98.00]
Hsieh 2004	1	20	0	20	2.6%	3.00 [0.13, 69.52]
Lai 2002	2	19	1	16	5.7%	1.68 [0.17, 16.91]
Lee 2009	1	26	0	24	2.7%	2.78 [0.12, 65.08]
Masi 1993	6	63	2	61	10.7%	2.90 [0.61, 13.84]
Nayak 2017	3	228	0	229	2.6%	7.03 [0.37, 135.34]
Segal 2003	6	81	1	83	5.2%	6.15 [0.76, 49.94]
Simons 1999	1	399	0	396	2.6%	2.98 [0.12, 72.87]
Simons 2003	8	42	12	43	62.3%	0.68 [0.31, 1.50]
**Subtotal (95% CI)**		**916**		**907**	**100.0%**	**1.87 [1.10, 3.17]**
Total events	33		16			
Heterogeneity: Chi^2 = 9.87, df = 9 (P = 0.36); I^2 = 9%						
Test for overall effect: Z = 2.32 (P = 0.02)						
**6.1.2 CTZ vs other AHs**						
Lai 2002	2	19	1	16	11.7%	1.68 [0.17, 16.91]
Lee 2009	1	11	0	9	5.9%	2.50 [0.11, 54.87]
Nayak 2017	3	228	0	220	5.5%	6.76 [0.35, 130.03]
Sienra-monge 1999	1	40	0	40	5.4%	3.00 [0.13, 71.51]
Tinkelman 1996	10	123	5	63	71.5%	1.02 [0.37, 2.87]
**Subtotal (95% CI)**		**421**		**348**	**100.0%**	**1.61 [0.72, 3.58]**
Total events	17		6			
Heterogeneity: Chi^2 = 1.87, df = 4 (P = 0.76); I^2 = 0%						
Test for overall effect: Z = 1.17 (P = 0.24)						
**6.1.3 CTZ vs montelukast**						
Chen 2006	1	20	0	20	50.0%	3.00 [0.13, 69.52]
Hsieh 2004	1	20	0	20	50.0%	3.00 [0.13, 69.52]
**Subtotal (95% CI)**		**40**		**40**	**100.0%**	**3.00 [0.33, 27.69]**
Total events	2		0			
Heterogeneity: Chi^2 = 0.00, df = 1 (P = 1.00); I^2 = 0%						
Test for overall effect: Z = 0.97 (P = 0.33)						

Test for subgroup differences: Chi^2 = 0.29, df = 2 (P = 0.86), I^2 = 0%

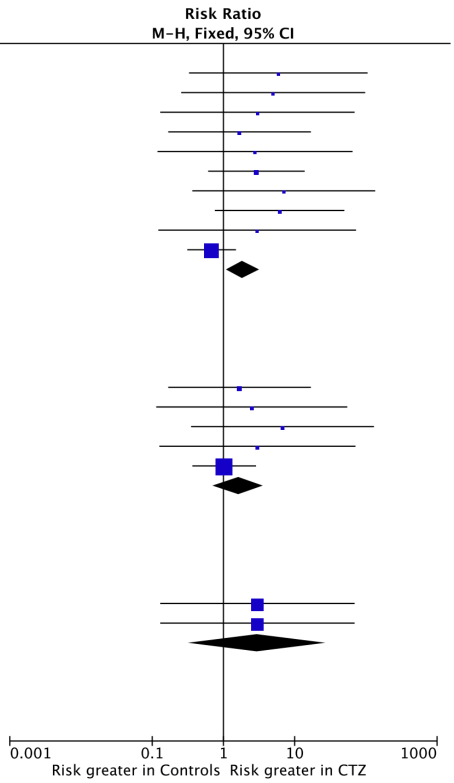

Supplementary File 5.3 Insomnia

Study or Subgroup	CTZ group Events	CTZ group Total	Control group Events	Control group Total	Weight	Risk Ratio M-H, Random, 95% CI
Allegra L 1993	1	54	1	53	6.6%	0.98 [0.06, 15.29]
Baelde 1992	3	46	2	46	13.9%	1.50 [0.26, 8.56]
Simons 1999	35	399	21	396	42.4%	1.65 [0.98, 2.79]
Simons 2003	9	42	17	43	37.0%	0.54 [0.27, 1.08]
**Total (95% CI)**		**541**		**538**	**100.0%**	**1.04 [0.49, 2.22]**
Total events	48		41			

Heterogeneity: Tau^2 = 0.28; Chi^2 = 6.60, df = 3 (P = 0.09); I^2 = 55%
Test for overall effect: Z = 0.11 (P = 0.91)

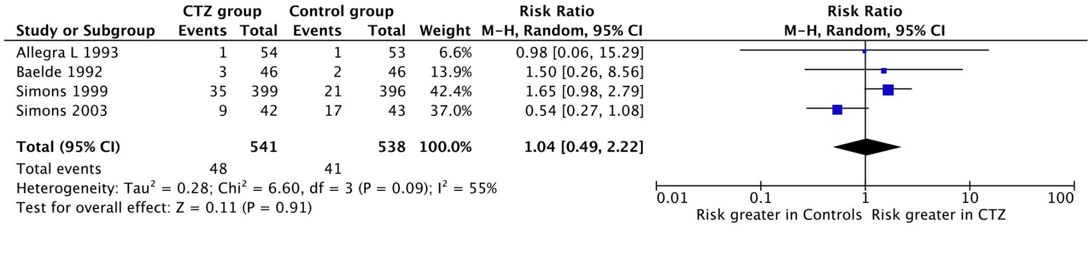

Supplementary File 5.4 Headache

Study or Subgroup	CTZ group Events	CTZ group Total	Control group Events	Control group Total	Weight	Risk Ratio M-H, Random, 95% CI
**7.1.1 CTZ vs placebo**						
Allegra L 1993	1	54	1	53	1.8%	0.98 [0.06, 15.29]
Baelde 1992	0	92	2	46	1.5%	0.10 [0.00, 2.06]
Hsieh 2004	0	20	1	20	1.4%	0.33 [0.01, 7.72]
Lai 2002	0	19	1	16	1.4%	0.28 [0.01, 6.51]
Masi 1993	2	63	1	61	2.4%	1.94 [0.18, 20.81]
Nayak 2017	8	228	7	229	13.5%	1.15 [0.42, 3.11]
Pearlman 1997	21	139	13	66	34.3%	0.77 [0.41, 1.44]
Segal 2003	7	81	8	83	14.4%	0.90 [0.34, 2.36]
Winder 1996	20	139	11	68	29.4%	0.89 [0.45, 1.75]
**Subtotal (95% CI)**		**835**		**642**	**100.0%**	**0.84 [0.58, 1.21]**
Total events	59		45			
Heterogeneity: Tau² = 0.00; Chi² = 3.69, df = 8 (P = 0.88); I² = 0%						
Test for overall effect: Z = 0.93 (P = 0.35)						
**7.1.2 CTZ vs other AHs**						
Nayak 2017	8	228	8	220	66.4%	0.96 [0.37, 2.53]
Tinkelman 1996	4	123	4	63	33.6%	0.51 [0.13, 1.98]
**Subtotal (95% CI)**		**351**		**283**	**100.0%**	**0.78 [0.36, 1.71]**
Total events	12		12			
Heterogeneity: Tau² = 0.00; Chi² = 0.56, df = 1 (P = 0.45); I² = 0%						
Test for overall effect: Z = 0.62 (P = 0.53)						
**7.1.3 CTZ vs montelukast**						
Hsieh 2004	1	20	1	20	100.0%	1.00 [0.07, 14.90]
**Subtotal (95% CI)**		**20**		**20**	**100.0%**	**1.00 [0.07, 14.90]**
Total events	1		1			
Heterogeneity: Not applicable						
Test for overall effect: Z = 0.00 (P = 1.00)						

Test for subgroup differences: Chi² = 0.05, df = 2 (P = 0.98), I² = 0%

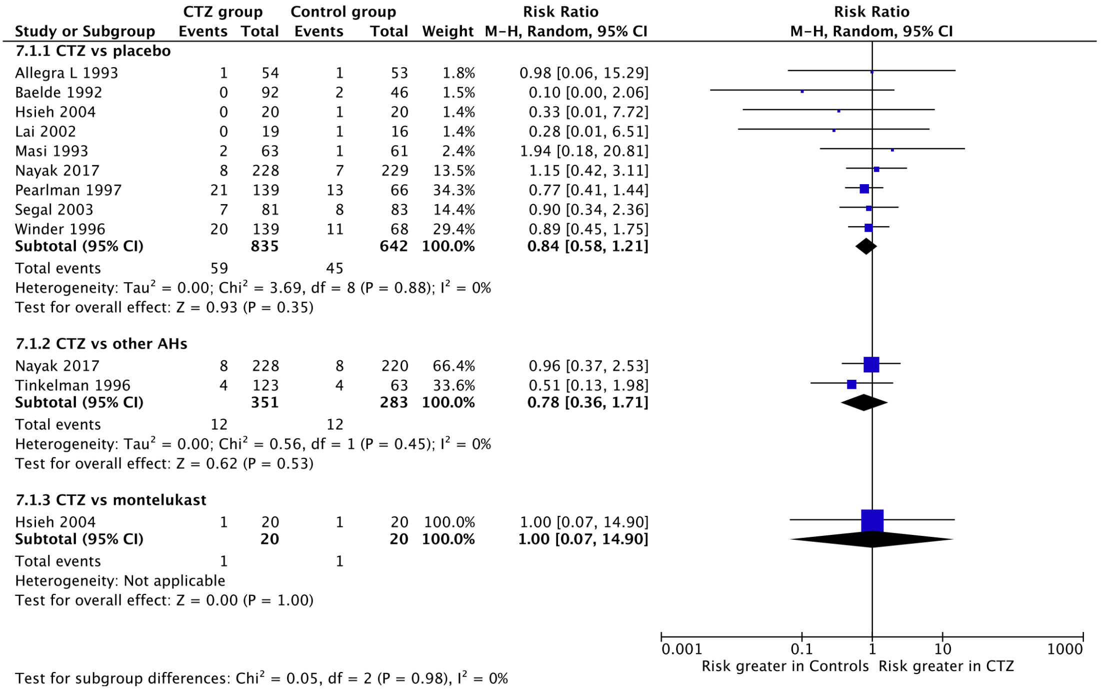

Supplementary File 5.5 Fatigue

Study or Subgroup	CTZ group Events	CTZ group Total	Control group Events	Control group Total	Weight	Risk Ratio M-H, Fixed, 95% CI
**8.1.1 CTZ vs placebo**						
Baelde 1992	2	92	1	46	15.9%	1.00 [0.09, 10.74]
Hsieh 2004	0	20	1	20	17.9%	0.33 [0.01, 7.72]
Lai 2002	1	19	0	16	6.4%	2.55 [0.11, 58.60]
Simons 1999	13	399	5	396	59.8%	2.58 [0.93, 7.17]
**Subtotal (95% CI)**		**530**		**478**	**100.0%**	**1.93 [0.84, 4.40]**
Total events	16		7			
Heterogeneity: Chi² = 1.83, df = 3 (P = 0.61); I² = 0%						
Test for overall effect: Z = 1.55 (P = 0.12)						
**8.1.2 CTZ vs other AHs**						
Lai 2002	1	19	0	16	7.2%	2.55 [0.11, 58.60]
Lee 2009	0	11	1	9	21.9%	0.28 [0.01, 6.10]
Tinkelman 1996	5	123	4	63	70.8%	0.64 [0.18, 2.30]
**Subtotal (95% CI)**		**153**		**88**	**100.0%**	**0.70 [0.24, 2.02]**
Total events	6		5			
Heterogeneity: Chi² = 1.02, df = 2 (P = 0.60); I² = 0%						
Test for overall effect: Z = 0.66 (P = 0.51)						

Test for subgroup differences: Chi² = 2.19, df = 1 (P = 0.14), I² = 54.2%

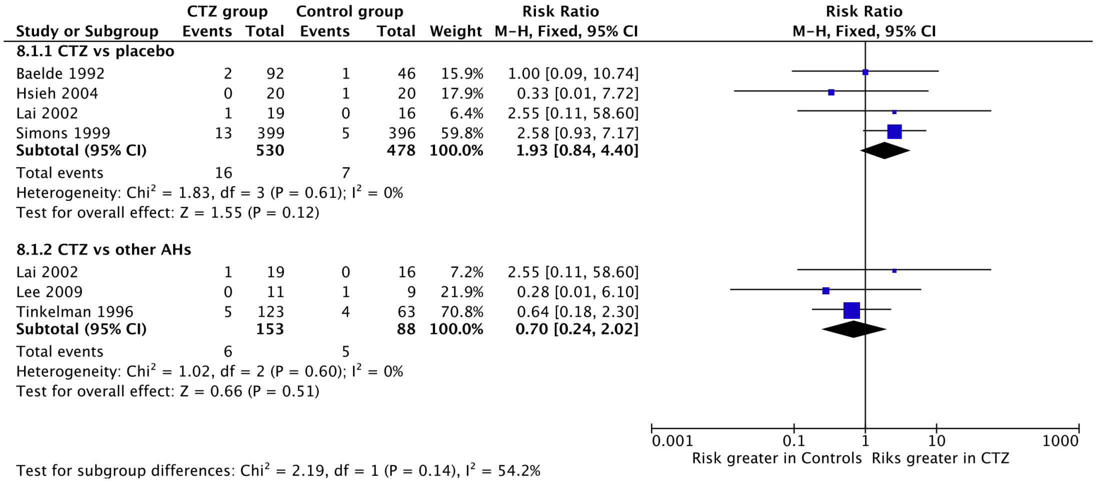

Supplementary File 5.6 Nausea

Study or Subgroup	CTZ group Events	Total	Control group Events	Total	Weight	Risk Ratio M-H, Random, 95% CI	Risk Ratio M-H, Random, 95% CI
**9.1.1 CTZ vs placebo**							
Lai 2002	0	228	2	229	42.8%	0.20 [0.01, 4.16]	
Segal 2003	3	81	1	83	57.2%	3.07 [0.33, 28.94]	
**Subtotal (95% CI)**		**309**		**312**	**100.0%**	**0.96 [0.07, 13.69]**	
Total events	3		3				
Heterogeneity: $Tau^2 = 1.92$; $Chi^2 = 2.04$, df = 1 (P = 0.15); $I^2 = 51\%$							
Test for overall effect: Z = 0.03 (P = 0.97)							
**9.1.2 CTZ vs other AHs**							
Lai 2002	0	19	1	16	25.5%	0.28 [0.01, 6.51]	
Nayak 2017	0	228	3	220	28.2%	0.14 [0.01, 2.65]	
Tinkelman 1996	4	123	1	63	46.3%	2.05 [0.23, 17.95]	
**Subtotal (95% CI)**		**370**		**299**	**100.0%**	**0.58 [0.11, 3.18]**	
Total events	4		5				
Heterogeneity: $Tau^2 = 0.40$; $Chi^2 = 2.42$, df = 2 (P = 0.30); $I^2 = 17\%$							
Test for overall effect: Z = 0.63 (P = 0.53)							

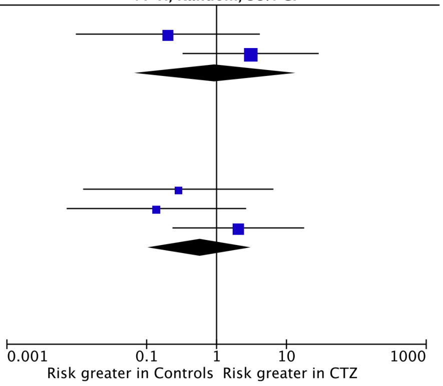

Test for subgroup differences: $Chi^2 = 0.10$, df = 1 (P = 0.76), $I^2 = 0\%$

Supplementary File 5.7 Abdominal pain

Study or Subgroup	CTZ group Events	Total	Control group Events	Total	Weight	Risk Ratio M-H, Fixed, 95% CI	Risk Ratio M-H, Fixed, 95% CI
**10.1.1 CTZ vs placebo**							
Allegra L 1993	1	54	2	53	7.0%	0.49 [0.05, 5.25]	
Baelde 1992	2	92	2	46	9.2%	0.50 [0.07, 3.44]	
Pearlman 1997	13	139	3	66	14.1%	2.06 [0.61, 6.97]	
Segal 2003	2	81	0	83	1.7%	5.12 [0.25, 105.07]	
Wahn 1998	17	398	17	397	58.8%	1.00 [0.52, 1.93]	
Winder 1996	8	139	2	68	9.3%	1.96 [0.43, 8.97]	
**Subtotal (95% CI)**		**903**		**713**	**100.0%**	**1.22 [0.75, 1.99]**	
Total events	43		26				
Heterogeneity: $Chi^2 = 3.70$, df = 5 (P = 0.59); $I^2 = 0\%$							
Test for overall effect: Z = 0.81 (P = 0.42)							
**10.1.2 CTZ vs other AHs**							
Tinkelman 1996	12	123	3	63	100.0%	2.05 [0.60, 7.00]	
**Subtotal (95% CI)**		**123**		**63**	**100.0%**	**2.05 [0.60, 7.00]**	
Total events	12		3				
Heterogeneity: Not applicable							
Test for overall effect: Z = 1.14 (P = 0.25)							

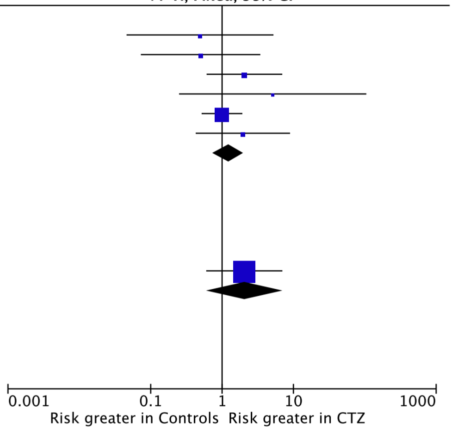

Test for subgroup differences: $Chi^2 = 0.58$, df = 1 (P = 0.45), $I^2 = 0\%$

Supplementary File 5.8 Diarrhea

Study or Subgroup	CTZ group Events	Total	Control group Events	Total	Weight	Risk Ratio M-H, Fixed, 95% CI	Risk Ratio M-H, Fixed, 95% CI
Allegra L 1993	1	53	1	54	25.0%	1.02 [0.07, 15.87]	
Simons 2003	3	42	3	43	75.0%	1.02 [0.22, 4.79]	
**Total (95% CI)**		**95**		**97**	**100.0%**	**1.02 [0.27, 3.93]**	
Total events	4		4				
Heterogeneity: $Chi^2 = 0.00$, df = 1 (P = 1.00); $I^2 = 0\%$							
Test for overall effect: Z = 0.03 (P = 0.97)							

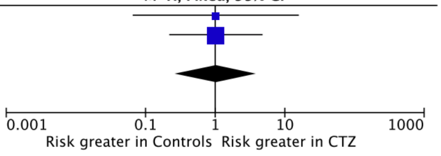

Supplementary File 5.9 Vomiting

Study or Subgroup	CTZ group Events	CTZ group Total	Control group Events	Control group Total	Weight	Risk Ratio M-H, Fixed, 95% CI
**11.1.1 CTZ vs placebo**						
Allegra L 1993	0	54	1	53	60.3%	0.33 [0.01, 7.86]
Nayak 2017	2	228	1	229	39.7%	2.01 [0.18, 22.00]
**Subtotal (95% CI)**		**282**		**282**	**100.0%**	**1.00 [0.17, 5.68]**
Total events	2		2			
Heterogeneity: $Chi^2 = 0.80$, df = 1 (P = 0.37); $I^2 = 0\%$						
Test for overall effect: Z = 0.01 (P = 1.00)						
**11.1.2 CTZ vs other LTD**						
Nayak 2017	2	228	4	220	100.0%	0.48 [0.09, 2.61]
**Subtotal (95% CI)**		**228**		**220**	**100.0%**	**0.48 [0.09, 2.61]**
Total events	2		4			
Heterogeneity: Not applicable						
Test for overall effect: Z = 0.85 (P = 0.40)						

Test for subgroup differences: $Chi^2 = 0.34$, df = 1 (P = 0.56), $I^2 = 0\%$

Risk Ratio M-H, Fixed, 95% CI

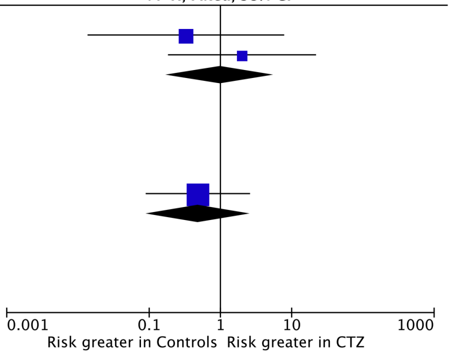

Supplementary File 5.10 Increased appetite

Study or Subgroup	CTZ group Events	CTZ group Total	Control group Events	Control group Total	Weight	Risk Ratio M-H, Fixed, 95% CI
Allegra L 1993	1	54	0	53	33.5%	2.95 [0.12, 70.72]
Simons 1999	2	399	1	396	66.5%	1.98 [0.18, 21.80]
**Total (95% CI)**		**453**		**449**	**100.0%**	**2.31 [0.34, 15.51]**
Total events	3		1			
Heterogeneity: $Chi^2 = 0.04$, df = 1 (P = 0.85); $I^2 = 0\%$						
Test for overall effect: Z = 0.86 (P = 0.39)						

Risk Ratio M-H, Fixed, 95% CI

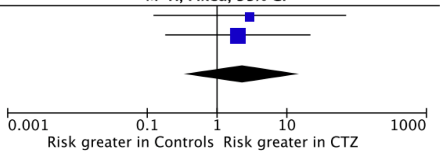

Supplementary File 5.11 Nervousness

Study or Subgroup	CTZ group Events	CTZ group Total	Control group Events	Control group Total	Weight	Risk Ratio M-H, Fixed, 95% CI
Allegra L 1993	2	54	1	53	3.6%	1.96 [0.18, 21.01]
Segal 2003	2	81	2	83	7.1%	1.02 [0.15, 7.10]
Simons 1999	5	399	7	396	25.3%	0.71 [0.23, 2.21]
Simons 2003	12	42	18	43	64.0%	0.68 [0.38, 1.24]
**Total (95% CI)**		**576**		**575**	**100.0%**	**0.76 [0.46, 1.26]**
Total events	21		28			
Heterogeneity: $Chi^2 = 0.85$, df = 3 (P = 0.84); $I^2 = 0\%$						
Test for overall effect: Z = 1.07 (P = 0.28)						

Risk Ratio M-H, Fixed, 95% CI

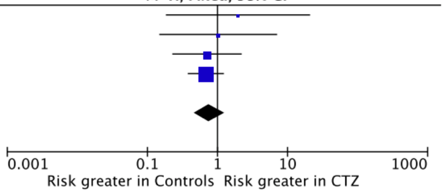

Supplementary File 6 GRADE Evidence Profile

Certainty assessment						Number of patients		Effect	Certainty
Number of studies	Risk of bias	Inconsistency	Indirectness	Imprecision	Publication bias	CTZ	Controls	(95% CI)	
3.3.1.1 Total symptom score									
at 1 week<br>2 RCTs	not serious	not serious	not serious	not serious	**serious**[d]	129	136	MD, -0.32<br>[-0.52, -0.12]	⊕⊕⊕◯<br>moderate
at 2 weeks<br>4 RCTs	not serious	**serious**[b]	not serious	not serious	**serious**[d]	449	411	MD, -0.25<br>[-0.35, -0.14]	⊕⊕◯◯<br>low
at 4 weeks<br>4 RCTs	not serious	not serious	not serious	not serious	**serious**[d]	65	60	MD, -4.07<br>[-4.71, -3.43]	⊕⊕⊕◯<br>moderate
at 8 weeks<br>4 RCTs	not serious	not serious	not serious	not serious	**serious**[d]	65	60	MD, -4.22<br>[-4.73, -3.72]	⊕⊕⊕◯<br>moderate
at 12 weeks<br>4 RCTs	not serious	not serious	not serious	not serious	**serious**[d]	65	60	MD, -5.63<br>[-6.14, -5.13]	⊕⊕⊕◯<br>moderate
3.3.1.3 PRQLQ									
CTZ vs placebo<br>3 RCTs	not serious	not serious	not serious	not serious	**serious**[d]	65	60	MD, -23.16<br>[-26.92, -19.39]	⊕⊕⊕◯<br>moderate

CTZ vs other AHs<br>2 RCTs	not serious	**serious**[b]	not serious	not serious	**serious**[d]	65	60	MD, -0.88<br>[-11.99, 10.22]	⊕⊕◯◯<br>low
3.4.1 Overall AEs									
CTZ vs placebo<br>7 RCTs	not serious	not serious	not serious	not serious	not serious	532	361	RR, 1.07<br>[0.85, 1.34]	⊕⊕⊕⊕<br>high
3.4.3-1 Somnolence									
CTZ vs placebo<br>10 RCTs	not serious	not serious	not serious	not serious	**serious**[d]	916	907	RR, 1.62<br>[1.02, 2.57]	⊕⊕⊕◯<br>moderate
CTZ vs other AHs<br>5 RCTs	not serious	not serious	not serious	not serious	**serious**[d]	421	348	RR, 1.61<br>[0.72, 3.58]	⊕⊕⊕◯<br>moderate
CTZ vs M<br>2 RCTs	not serious	not serious	not serious	**serious**[c]	**serious**[d]	40	40	RR, 3.00<br>[0.33, 27.69]	⊕⊕◯◯<br>low
3.4.3-2 Insomnia									
CTZ vs placebo<br>4 RCTs	not serious	**serious**[b]	not serious	not serious	not serious	541	538	RR, 1.04<br>[0.49, 2.22]	⊕⊕⊕◯<br>moderate
3.4.3-3 Headache									
CTZ vs placebo<br>9 RCTs	not serious	not serious	not serious	not serious	**serious**[d]	835	642	RR, 0.84<br>[0.58, 1.21]	⊕⊕⊕◯<br>moderate

CTZ vs other AHs<br>2 RCTs	**serious**[a]	not serious	not serious	not serious	not serious	351	283	RR, 0.78<br>[0.36, 1.71]	⊕⊕◯◯<br>low
3.4.3-4 Fatigue									
CTZ vs placebo<br>4 RCTs	not serious	not serious	not serious	not serious	**serious**[d]	530	478	RR, 1.93<br>[0.84, 4.40]	⊕⊕⊕◯<br>moderate
CTZ vs other AHs<br>3 RCTs	**serious**[a]	not serious	not serious	not serious	not serious	153	88	RR, 0.70<br>[0.24, 2.02]	⊕⊕◯◯<br>low
3.4.4-1 Nausea									
CTZ vs placebo<br>2 RCTs	not serious	**serious**[b]	not serious	**serious**[c]	not serious	530	478	RR, 0.96<br>[0.07, 13.69]	⊕⊕◯◯<br>low
CTZ vs other AHs<br>2 RCTs	**serious**[a]	not serious	not serious	not serious	not serious	370	299	RR, 0.58<br>[0.11, 3.18]	⊕⊕⊕◯<br>moderate
3.4.4-2 Abdominal pain									
CTZ vs placebo<br>6 RCTs	not serious	not serious	not serious	not serious	not serious	903	713	RR, 1.22<br>[0.75, 1.99]	⊕⊕⊕⊕<br>high
3.4.4-3 Diarrhea									
CTZ vs placebo<br>2 RCTs	not serious	not serious	not serious	**serious**[c]	not serious	95	97	RR, 1.02<br>[0.27, 3.93]	⊕⊕⊕◯<br>moderate

3.4.4-4 Vomiting

CTZ vs placebo 2 RCTs	not serious	not serious	not serious	**serious**[c]	not serious	282	282	RR, 1.00 [0.17, 5.68]	⊕⊕⊕◯ moderate

3.4.4-5 Increased appetite

CTZ vs placebo 2 RCTs	not serious	not serious	not serious	**serious**[c]	not serious	453	449	RR, 2.31 [0.43, 15.51]	⊕⊕⊕◯ moderate

3.4.5 Nervousness

CTZ vs placebo 4 RCTs	not serious	not serious	not serious	not serious	**serious**[d]	576	575	RR, 0.76 [0.46, 1.26];	⊕⊕⊕◯ moderate

CTZ: cetirizine; M: montelukast; CI: confidence interval; RR: risk ratio

GRADE Working Group grades of evidence

High certainty: We are very confident that the true effect lies close to that of the estimate of the effect.

Moderate certainty: We are moderately confident in the effect estimate; the true effect is likely to be close to the estimate of effect, but there is a possibility that it is substantially different.

Low certainty: Our confidence in the effect estimate is limited; the true effect may be substantially different from the estimate of the effect.

Very low certainty: We have very little confidence in the effect estimate; the true effect is likely to be substantially different from the estimate of effect.

a Downgraded due to high risk of bias

b Downgraded due to unexplained heterogeneity ($I^2 > 50\%$)

c Downgraded due to wide confidence interval as a binary outcome

d Downgraded due to potential publication bias
